# Association of poultry vaccination with interspecies transmission and molecular evolution of H5 subtype avian influenza virus

**DOI:** 10.1126/sciadv.ado9140

**Published:** 2025-01-22

**Authors:** Bingying Li, Jayna Raghwani, Sarah C. Hill, Sarah François, Noémie Lefrancq, Yilin Liang, Zengmiao Wang, Lu Dong, Phillipe Lemey, Oliver G. Pybus, Huaiyu Tian

**Affiliations:** ^1^ State Key Laboratory of Remote Sensing Science, National Key Laboratory of Intelligent Tracking and Forecasting for Infectious Diseases, Beijing Research Center for Respiratory Infectious Diseases, School of National Safety and Emergency Management, Center for Global Change and Public Health, Beijing Normal University, Beijing, China.; ^2^ Department of Pathobiology and Population Sciences, The Royal Veterinary College, London, UK.; ^3^ Department of Biology, University of Oxford, Oxford, UK.; ^4^ UMR DGIMI, University of Montpellier, INRAE, Montpellier, France.; ^5^ Department of Genetics, University of Cambridge, Cambridge, UK.; ^6^ Ministry of Education Key Laboratory for Biodiversity and Ecological Engineering, College of Life Sciences, Beijing Normal University, Beijing, China.; ^7^ Department of Microbiology, Immunology and Transplantation, Rega Institute, Clinical and Epidemiological Virology, KU Leuven, 3000 Leuven, Belgium.

## Abstract

The effectiveness of poultry vaccination in preventing the transmission of highly pathogenic avian influenza viruses (AIVs) has been debated, and its impact on wild birds remains uncertain. Here, we reconstruct the movements of H5 subtype AIV lineages among vaccinated poultry, unvaccinated poultry, and wild birds, worldwide, from 1996 to 2023. We find that there is a time lag in viral transmission among different host populations and that movements from wild birds to unvaccinated poultry were more frequent than those from wild birds to vaccinated poultry. Furthermore, our findings suggest that the HA (hemagglutinin) gene of the AIV lineage that circulated predominately in Chinese poultry experienced greater nonsynonymous divergence and adaptive fixation than other lineages. Our results indicate that the epidemiological, ecological, and evolutionary consequences of widespread AIV vaccination in poultry may be linked in complex ways and that much work is needed to better understand how such interventions may affect AIV transmission to, within, and from wild birds.

## Teaser

Mass poultry vaccination affects H5 avian influenza virus transmission and evolution, revealing complex host-virus dynamics.

## INTRODUCTION

During the summer of 2022, seabirds in many European, North American, and African countries suffered unprecedented mortality from avian influenza virus (AIV) (*1*). The causative virus is highly pathogenic H5 subtype AIV (HPAIV) belonging to clade 2.3.4.4b and has been detected at high incidences in wild birds (*2*). Wild birds, the natural reservoir of AIVs, can acquire and transmit viruses to poultry or mammals (*3*, *4*) and play a major role in the maintenance and global dissemination of AIVs (*5*–*8*). In addition, transmission within and between species of genetically diverse AIVs can facilitate genomic reassortment (*9*, *10*) and the emergence of previously unidentified HPAIV lineages (*11*–*16*). Consequently, HPAIV pose a severe threat to human, wild, and domestic animal health.

**Fig. 1.**
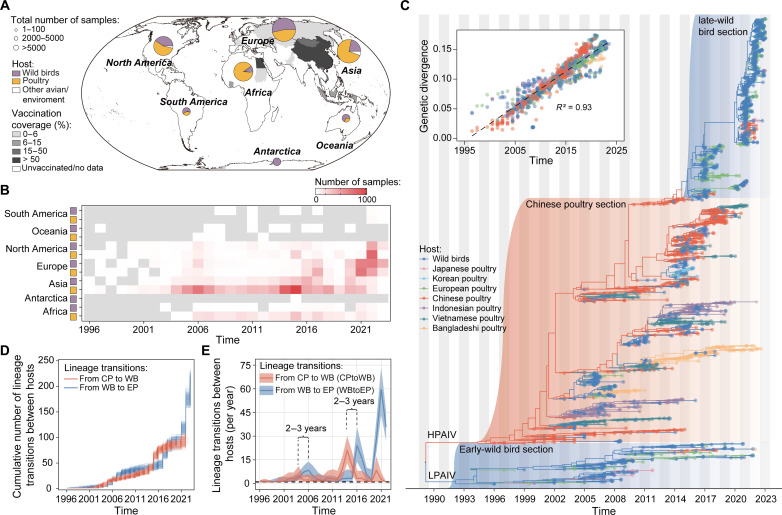
Global dynamics of H5 AIV. (**A**) Vaccination coverage and number of H5 AIV HA gene sequences across different continents and countries. Countries are shaded according to the vaccination coverage in poultry in 2010. The pie charts show the total number of H5 AIV HA gene sequences sampled from poultry, wild birds, and environment since 1996. Viral sequences are categorized according to their host: wild birds (purple), poultry (yellow), and other avian/environment (white). (**B**) Number of H5 AIV HA gene sequences sampled in poultry and wild birds, per year, per continent. (**C**) Maximum clade credibility tree of H5 AIV HA gene sequences sampled from 1996 to 2023. Tree tips are colored according to the host from which the sequence was sampled, whereas internal branches represent ancestral host states inferred using the asymmetric discrete phylogenetic model (dark blue, wild birds; orange, Chinese poultry; light green, European poultry; purple, Indonesian poultry; pink, Japanese poultry; light blue, Korean poultry; dark green, Vietnamese poultry; and yellow, Bangladeshi poultry). HPAIV, highly pathogenic influenza virus; LPAIV, lowly pathogenic influenza virus. Inset: a root-to-tip regression of genetic divergence against dates of sample collection. (**D**) Cumulative number of host population changes (Markov jumps) on lineages in the HA gene phylogeny. The lineage transitions between hosts (wild birds, WB; Chinese poultry, CP; and European poultry, EP) were summarized from a posterior sample of trees from the asymmetric discrete phylogenetic model. (**E**) Time series of the annual mean number of HA gene lineage transitions between wild birds, Chinese poultry, and European poultry.

To protect poultry from HPAIV infection, some countries, mainly in Asia and Africa, have implemented vaccination programs in poultry for H5 subtype HPAIV (Fig. 1A) (*17*–*20*). On the basis of national vaccination data from 2010, Egypt had the highest vaccination coverage in poultry (82%), followed by China [73% in 2009 and 93% in layer and slow-growing meat chickens, 14% in fast-growing meat chickens, and <30% in ducks in 2018 (*21*)], Vietnam (31%), and Indonesia (12%) (*22*). In 2013, the vaccination coverage of commercial layer flocks in two districts of Bangladesh was reported to be 32 and 54% (*23*). France and the Netherlands have also vaccinated poultry; however, their overall vaccination coverages are very low (<0.1%). Since 2005, China has implemented a nationwide vaccination program (*24*) and is responsible for >90% of the global consumption of H5 AIV vaccines. Because of the continuing evolution of H5 AIV, vaccine strains are frequently updated to ensure their effectiveness (table S1) (*25*, *26*). Several studies have suggested that extensive vaccination of Chinese poultry against H5 AIV has suppressed outbreaks effectively and decreased substantially the prevalence of H5 AIV in live bird markets (*23*, *26*–*28*).

However, there are concerns that mass vaccination against H5 AIV could affect the molecular evolution of the virus (*29*). For example, after the introduction of mass vaccination against H5N1 AIV in China, a notable increase in the evolutionary rate of H5N1 AIV was observed during 2005 to 2010 (*30*). Similarly, in North America, the evolutionary rate of H5N2 AIV in Mexico was inferred to be higher between 1993 and 2002 after a period of mass vaccination in this region, compared to the evolutionary rate of H5 AIV in the United States, where vaccination was not used (*31*). Furthermore, the AIV lineages that circulate in Egypt and Indonesia, where vaccination against H5N1 is prevalent, are characterized by higher evolutionary rates and a greater number of positively selected sites in the hemagglutinin (HA) gene compared to countries where vaccination is absent, such as Nigeria, Turkey, and Thailand (*32*, *33*). These studies have concentrated mainly on viruses sampled from poultry and lack data from wild birds and at the wild bird–poultry interface, which hinders our understanding of the underlying mechanisms of AIV evolution.

Here, we investigate the evolutionary history of H5 subtype AIV lineages among vaccinated poultry, unvaccinated poultry, and wild birds, worldwide, from 1996 to 2023 and compare the evolutionary dynamics of H5 AIV in different host populations that vary in vaccination status. Our results can be used to inform the development of effective strategies for AIV vaccination and immunization.

## RESULTS

### Interspecies transmission of H5 AIV at the interface of poultry and wild birds

We collated a total of 22,606 HA gene sequences belonging to H5 AIV, sampled from each continent since 1996 (Fig. 1, A and B). The virus sequences were unevenly distributed over time and space across host populations (table S2). Therefore, for downstream analyses, we focused on Europe and Asia, from where sufficient viral genetic data were available from long-term sampling of poultry and wild birds to quantify the dynamics of virus transmission and evolution within and among host populations. By taking into account the implementation of vaccination programs and the availability of viral genetic sequences (fig. S1), we categorized sequences into eight groups based on the country of sampling, host species, and vaccination status: wild birds (without vaccination), European poultry (without vaccination), Japanese poultry (without vaccination), Korean poultry (without vaccination), Indonesian poultry (with vaccination), Bangladeshi poultry (with vaccination), Vietnamese poultry (with vaccination), and Chinese poultry (with vaccination). For the remainder of the analysis, we use the terms with vaccination and without vaccination as a simplified binary classification while acknowledging that nationally reported vaccination coverages can range continuously from 0 to 100% and coverage can vary within each country.

To investigate H5 AIV transmission and evolution between and within these eight groups since 1996, we used 2407 H5 HA gene sequences to reconstruct time scaled phylogenies under an asymmetric discrete trait model (Fig. 1C, fig. S2, and table S3). Interspecies movements of H5 AIV lineages from Chinese poultry to wild birds and from wild birds to European poultry were frequently observed (Fig. 1D). Bayesian reconstruction of the host status of H5 AIV lineages indicates that there were three waves of spillovers from Chinese poultry to wild birds (CPtoWB) and from wild birds to European poultry (WBtoEP) (Fig. 1E). To investigate lineage transitions among these three host types before 2020, we conducted an extended convergent cross mapping (CCM) analysis on the time series of mean annual lineage transitions (Markov jumps) from Chinese poultry to wild birds and from wild birds to European poultry, between 1996 and 2019. We find that CPtoWB transitions preceded WBtoEP transitions, with a time lag of ~2 to 3 years (Fig. 1E and fig. S3). The observed pattern is robust to different genomic sampling strategies (figs. S4 to S7).

**Fig. 2.**
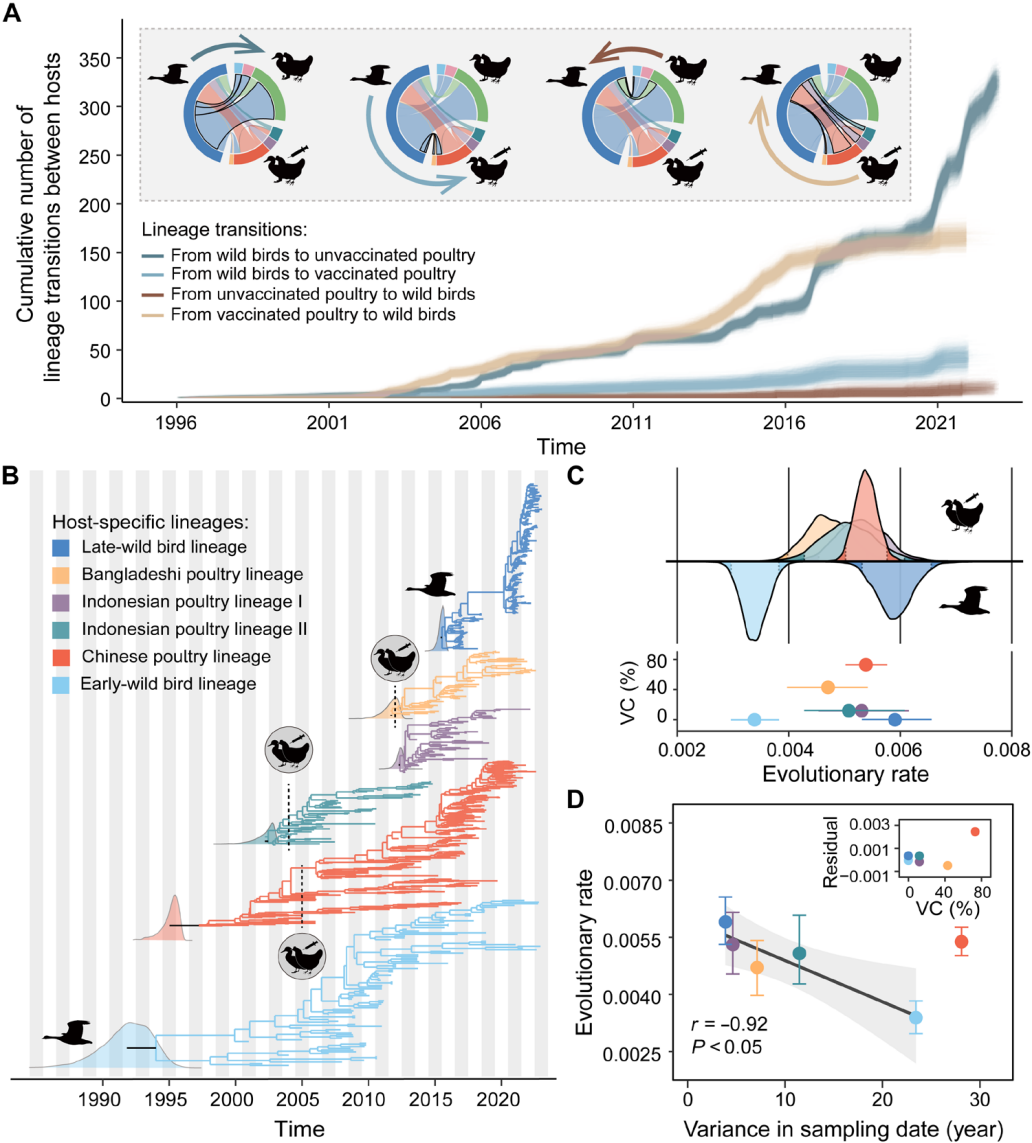
Transmission and evolution of the HA gene of H5 AIV between and within different host populations. (**A**) Interspecies lineage transmission between wild birds and poultry populations with different vaccination statues. The plots were summarized from a posterior sample of trees from the asymmetric discrete phylogenetic model. Chord diagrams show the mean cumulative lineage transitions between different groups of sequences (dark blue, wild birds; orange, Chinese poultry; light green, European poultry; purple, Indonesian poultry; pink, Japanese poultry; light blue, Korean poultry; dark green, Vietnamese poultry; and yellow, Bangladeshi poultry). Animal silhouettes are from PhyloPic.org. (**B**) Time-resolved phylogenies of H5 AIV lineages in wild birds and vaccinated poultry. The dashed line represents the date when each country started implementing avian influenza vaccination for poultry. The density plot shows the estimated most recent common ancestor (tMRCA) for each lineage. (**C**) The estimated substitution rates of the HA gene vary among host-specific H5 AIV lineages. Lineages in unvaccinated host populations (the early- and late-wild bird lineages) are shown below the line, and those in vaccinated host populations (the Chinese, Bangladeshi, and Indonesian poultry lineages) are shown above the line. The highlighted region shows 95% CIs. The bottom panel shows a scatterplot of the vaccination coverage (VC%) associated with each lineage versus the inferred evolutionary rate for that lineage. (**D**) Scatterplot of the variance in sampling date versus estimated evolutionary rate, for each host-specific lineage. The error bars show the 95% CIs for estimated evolutionary rates. A regression analysis (excluding the Chinese poultry lineage data point) was conducted to show the negative relationship between expected due to the time dependency of inferred evolutionary rates (*r* = −0.92; *P* < 0.05) and highlight that the Chinese poultry data point is an outlier. The inset panel shows a scatterplot of vaccination coverage (VC%) associated with each lineage against the residual of the fitted regression.

Overall, more viral lineage transitions were observed from wild birds to unvaccinated poultry populations than to vaccinated poultry populations (Fig. 2A, fig. S8, and table S4). Interspecies transitions from unvaccinated poultry to wild birds were frequent after 2016 (fig. S8). Virus lineage movements between poultry populations were not common. This observation was also robust to diverse genomic sampling strategies (fig. S9 and table S5).

### Evolutionary dynamics of H5 AIV in different host populations

We next investigated the evolutionary dynamics of H5 AIV in wild birds and poultry populations with different vaccination levels. Poultry-dominated viral lineages were identified only for China (vaccinated since 2005), Bangladesh (vaccinated since 2012), and Indonesia (vaccinated since 2004) (Fig. 2B). Our results indicated that the Chinese poultry lineage had a higher substitution rate (mean rate = 5.38 × 10^−3^ sub per site per year; 95% highest posterior density (HPD): 5.02 × 10^−3^ to 5.76 × 10^−3^; *P* < 0.05) than the early-wild bird lineage (3.39 × 10^−3^ sub per site per year; 95% HPD: 2.97 × 10^−3^ to 3.83 × 10^−3^) (Fig. 2C). Notably, this result is robust to the time dependence of virus evolutionary rates (*34*) because both lineages have similarly high variation in sequence sampling dates (Fig. 2D). The substitution rates of the poultry-associated viral lineages in Bangladesh and Indonesia were also faster than those of the early-wild bird lineage (*P* < 0.05). However, there is no clear relationship between the substitution rate and vaccination coverage (Fig. 2C). In addition, the substitution rate of the Chinese poultry lineage during the vaccination period (5.12 × 10^−3^ sub per site per year; 95% HPD: 4.71 × 10^−3^ to 5.55 × 10^−3^) was faster than before vaccination (4.79 × 10^−3^ sub per site per year; 95% HPD: 4.38 × 10^−3^ to 5.18 × 10^−3^; *P* < 0.05; fig. S10). Furthermore, the substitution rate of the late-wild bird lineage was faster compared to the Chinese poultry lineage from which it emerged (5.91 × 10^−3^ sub per site per year; 95% HPD: 5.03 × 10^−3^ to 6.56 × 10^−3^). However, this result should be interpreted with caution as the late-wild bird lineage is associated with limited temporal sampling and therefore may harbor mildly deleterious mutations.

If the higher evolutionary rate in the HA gene in vaccinated poultry lineages is simply due to increased viral transmission facilitated by high poultry densities, then we should also observe higher evolutionary rates in other gene segments (*35*). In contrast, we estimate a faster rate of evolution for PB2 in the wild bird lineage (3.62 × 10^−3^ sub per site per year; 95% HPD: 3.23 × 10^−3^ to 3.92 × 10^−3^; *P* < 0.05) than in the Chinese poultry lineage (3.44 × 10^−3^ sub per site per year; 95% HPD: 3.08 × 10^−3^ to 3.78 × 10^−3^; figs. S11 and S12). These results suggest that the elevated evolutionary rate in the HA gene in Chinese poultry may not be attributable to higher transmission rates alone and instead could result from vaccine-driven selection in the HA gene.

In addition, we subdivided the poultry population into different species groups: chicken, duck, goose, and other (unclassified poultry). We found that domestic ducks maintained the spread of H5 AIV in poultry in China and Bangladesh (fig. S13, A and B), whereas domestic chickens were more important in H5 AIV transmission in Indonesian poultry (fig. S13, C and D). However, frequent interspecies transmission makes it difficult to identify specific poultry lineages.

### Viral adaptive evolution in host populations with different vaccination status

**Fig. 3.**
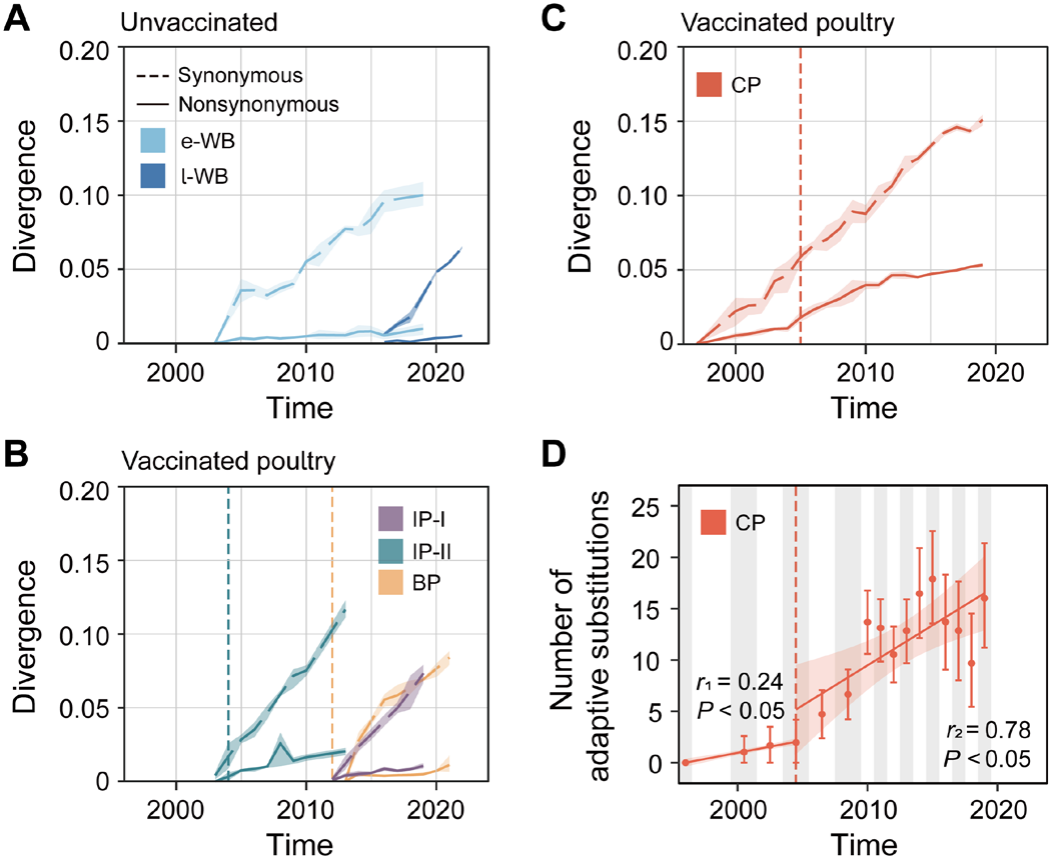
Temporal dynamics in divergence and adaptive fixation of the H5 AIV HA gene, in different host-specific lineages. (**A** to **C**) Nonsynonymous (solid lines) and synonymous (dashed lines) divergence of the HA gene through time. The host-specific lineages were classified into three groups: (A) early-wild bird lineage (e-WB; only the top lineage was retained) and late-wild bird linage (l-WB); (B) Indonesian poultry lineage I (IP-I), Indonesian poultry lineage II (IP-II), and Bangladeshi poultry lineage (BP); and (C) Chinese poultry lineage (CP). Divergences were computed using 1-year sliding windows. Shaded regions show 95% CIs. The dashed line represents the date when each country started implementing avian influenza vaccination for poultry. (**D**) Accumulation of viral adaptive substitutions in the Chinese poultry lineage. Two regression lines were estimated, before (*r*_1_) and after (*r*_2_) 2005. The first sequence, sampled in 1996, was used as the ancestral sequence, and those sampled from 1997 to 1999 were excluded due to insufficient sample size.

We then investigated whether the higher rate of molecular evolution observed in the Chinese poultry lineage, compared to the early-wild bird lineage, could be explained by a greater viral adaptive evolution in poultry. For each lineage, we calculated the nonsynonymous and synonymous divergence of the HA gene over time, from a known reference sequence [National Center for Biotechnology Information (NCBI) Reference Sequence: AF144305; Fig. 3, A to C]. Our results indicate higher nonsynonymous divergence for the Chinese poultry lineage than for other lineages, such as wild bird lineages and poultry lineages in Bangladesh and Indonesia, suggesting that the overall higher rate for Chinese lineage is associated with adaption rather than merely faster transmission (Chinese poultry lineage gradient = 0.0027, *P* < 0.05; early-wild bird lineage = 0.0005, *P* < 0.05; late-wild bird lineage = 0.0007, *P* < 0.05; Bangladeshi poultry lineage = 0.0009, *P* < 0.05; Indonesian poultry lineage I = 0.0011, *P* < 0.05; Indonesian poultry lineage II = 0.0021, *P* < 0.05). Furthermore, the nonsynonymous divergence rate increased in the Chinese poultry lineage after mass vaccination [gradient before 2005 = 0.0017, 95% confidence interval (CI) = 0.0011 to 0.0023; gradient between 2005 and 2010 = 0.0046, 95% CI = 0.0041 to 0.0052; table S6]. To test for the potential impact of different sampling intensities among countries, the Chinese poultry lineage was resampled and the results remained consistent (fig. S14). This indicates that the Chinese poultry lineage exhibited a greater nonsynonymous divergence, especially after vaccination, compared to the wild bird lineages and the poultry lineages that experienced lower vaccination coverage.

Because nonsynonymous divergence can result from either positive selection or random genetic drift, we also used an independent population genetic method to estimate the rate of adaptive substitutions in the HA gene of the Chinese poultry lineage. The estimated rate of adaptive fixation in that lineage was higher after the initiation of vaccination in Chinese poultry (gradient before 2005 = 0.24 adaptive fixations per codon per year; *P* < 0.05; gradient after 2005 = 0.78 adaptive fixations per codon per year; *P* < 0.05; Fig. 3D). These estimated adaptation fixation rates are rapid but lower than those previously inferred for the HA gene of human influenza subtype H3N2 (1.52) and subtype H1N1 (1.02) (*36*). Next, we estimated the *dN/dS* values for each host-associated lineage using the renaissance counting method (*37*). The mean *dN/dS* value is higher in the Chinese poultry lineage (0.24) than in the early-wild bird lineage (0.16) and the late-wild bird lineage (0.18) (table S7). The mean *dN/dS* values of the Bangladeshi (0.17) and Indonesian poultry I and II lineages (0.18 for both) were also comparatively lower.

Furthermore, *dN/dS* values were calculated for individual codons in the HA gene, for each host-specific H5 AIV lineage. We used multiple methods to detect sites under positive selection (Table 1). Our results indicate that the number of amino acid sites under positive selection in the Chinese poultry lineage was higher compared to the two wild bird lineages, as well as the Bangladeshi and Indonesian poultry lineages (Table 1). A higher number of positively selected sites were also observed in resampled Chinese poultry lineage (table S8), indicating that the finding is robust to diverse sampling strategies (tables S9 and S10).

**Table 1.** Positively selected sites in the HA gene among different host-specific H5 AIV lineages. FEL, Fixed Effects Likelihood; SLAC, Single Likelihood Ancestor Counting; FUBAR, Fast Unconstrained Bayesian AppRoximation; RC, renaissance counting method implemented in BEAST.

Host-specific lineages	Vaccination coverage	Livestock density in 2015 (birds/km^2^)^†^		Methods			
		Chicken	Duck	RC	FEL (*P* < 0.1)	SLAC (*P* < 0.1)	FUBAR (PP > 0.9)
Chinese poultry lineage	73% (*22*)	860	166	3, 61, 142, 156, 157, 171, 172, 178, 185, 205, 242, 285, 289, 527	87, 142*, 154*, 156, 157*, 172*, 185, 285*, 291*, 325*	3, 142*, 154*, 157*, 172*, 185*, 285*	142*, 154*, 157*, 171, 172*, 285*
Bangladesh poultry lineage	43% (*23*)	1917	387	170, 204, 205	204*, 205*	204, 205	170, 204*, 205*
Indonesia poultry lineage I	12% (*22*)	1512	121	170, 204, 211	205*, 554	205	170*, 205*
Indonesia poultry lineage II				10, 156, 529	3, 11, 156*, 529	156*	5*, 11*, 156*
Early-wild bird lineage	–	–		10, 102, 170, 171	102*, 170*, 171	102*, 170	102*, 170*, 171
Late-wild bird lineage	–	–		10	185, 507	-	252

*Sites inferred to be positively selected with a posterior probability (PP) of >0.95 or a *P* value (*P*) of <0.05.

†Mean livestock density was calculated using data from the Gridded Livestock of the World (*96*) website, hosted by the Food and Agriculture Organization (https://dataverse.harvard.edu/dataverse/glw). Regions with <10 birds/km^2^ were excluded from the calculation.

Most of the positively selected sites were associated with known immune epitopes. In the Chinese poultry lineage, these were mainly located in the receptor binding subdomain (fig. S15). For example, positive selection was observed at sites 156, 157, and 242, which are recognized as CD8^+^ T cell epitopes (*38*), and at site 87, which is associated with antibody epitope 65C6 (*39*). Within the late-wild bird lineage, positively selected site 252 is associated with the H5_246-260_ epitope, which can induce T cell activation in chickens immunized against H5 HA antigen (*40*). The positive selection analysis also revealed mutations (mostly in the receptor binding subdomain) that are associated with changes within the Chinese poultry lineage (D142E, H154Q/L/N, R156V/T/M/K/N/A, S157P/A, S171D/N, Q185R/K/S/G, and L285V/M/I) and with the transition of the virus from the Chinese poultry lineage to the late-wild bird lineage (E142, Q154, A/T156, P157, D171, R185, and V285). The amino acid states of these sites in the early-wild bird lineage (D142, N154, R156, S157, N171, Q185, and L285) are mostly different from those in the late-wild bird lineage, indicating that these changes are not reversion mutations.

## DISCUSSION

Since 2020, H5 subtype AIVs have caused outbreaks in European and Asian countries, posing a serious real threat to the poultry industry and a potential threat to public health. We reconstructed the interspecies transmission history of H5 subtype AIVs among wild birds and poultry populations with different vaccination levels. Our analysis reveals a shift from a lineage circulating within Chinese poultry to one circulating among wild birds. The wild bird lineage has been transmitted frequently to unvaccinated European poultry, whereas the spillover from wild birds to vaccinated poultry appears to be impeded. Furthermore, the virus lineage circulating in Chinese poultry exhibits evidence of more nonsynonymous and adaptive molecular evolution in the HA gene after the date of introduction of mass poultry vaccination. The Chinese poultry lineage may have experienced more vaccine-driven selection compared to other lineages. Although this pattern is consistent with the hypothesis that vaccination may have affected upon HA evolution in these poultry lineages, our finding does not establish a causal relationship and further virological work will be needed to test this hypothesis directly. In addition, more research is also needed to assess whether these mutations in HA have had an impact on the late-wild bird lineage.

Because of the higher virus prevalence, Chinese poultry, as well as Southeast Asian poultry, have been regarded as the main reservoirs of H5 HPAIV (*41*). H5 AIV spread to other regions through bird migration (*42*) and poultry trade (*43*), and our results reveal a 2- to 3-year delay between the peak of lineage dissemination from Chinese poultry and the peak of lineage introduction into European poultry. Although it has been suggested that the intercontinental spread of H5 AIV can occur within a single avian migratory cycle (*5*, *7*, *42*), the delay we observe may be attributed in part to preexisting immunity in wild birds, which may provide partial protection against infection and disease (*44*–*46*), leading to reduced circulation and potentially dampening large-scale outbreaks in wild birds. However, the duration of protection conferred by previous AIV infection and its impact on the epidemiology of AIV have not yet been fully elucidated (*47*). Furthermore, the relatively short generation length of many wild birds (~3 years) may result in higher turnover (*48*) of immunologically naïve wild birds in nature. As the reservoir of H5 AIV shifted from Chinese poultry to wild birds, frequent migration and large-scale spatial distribution of wild birds probably facilitated interspecies transmission between wild birds and poultry populations (*42*). It is crucial that countries and regions enhance regular surveillance of AIVs in wild birds and actively and closely monitor the dynamics of virus transmission.

Following the mass poultry vaccination strategy implemented in China since 2005, the spread of H5 AIV there has been relatively well controlled. Interspecies transmission of these viruses from or to Chinese poultry seems to be limited. However, the Chinese poultry lineage may have experienced more antigenic evolution compared to other lineages. Mutations at amino acid positions 136, 142, 157, 172, 201, and 205 in the H5 AIV HA gene have been shown to reduce reactivity to specific antibodies (*49*). Amino acids at positions 142, 172, and 205 also appear to function as immunodominant epitopes in H5 viruses (*49*). We detected positive selection at positions 142, 157, 172, and 205 in the Chinese poultry lineage but not in the wild bird lineages or the other poultry lineages. The H5N6 virus now circulating in Chinese poultry is antigenically divergent from the strains included in the commercial vaccine in China (*50*, *51*), which may result in reduced vaccine effectiveness. Our study indicates that, when vaccination is used, regular monitoring and refinement of vaccines to target emerging escape variants are necessary to respond to the emergence of previously unidentified viruses. Although contemporary H5 HPAIVs are considered unlikely to acquire the ability to infect and circulate stably among the human population (*52*), there is still an urgent need to control the spread of the virus among wild birds, not only for the preservation of wildlife but also for ensuring the safety of poultry (*53*).

Because H5 AIV has become increasingly widespread in wild bird populations (*54*, *55*), many unvaccinated countries may be considering large-scale vaccination of poultry populations to prevent the transmission of H5 AIV from wild birds to poultry. Achieving high vaccine coverage and efficacy will be critical for reducing the infection rates of H5 AIV in vaccinated populations (*56*–*61*). Studies have shown that inadequate vaccination coverage and suboptimal vaccine efficacy can lead to reemergence of H5 AIV outbreaks in vaccinated poultry (*62*–*64*), potentially allowing virulent strains to emerge and circulate within the population by keeping hosts alive while still allowing transmission (*65*, *66*). However, among the countries now vaccinating poultry against H5 AIV, China is, to the best of our knowledge, the only country that updates vaccine strains every 1 to 2 years. In contrast, other countries do not update their vaccine strains as frequently, which may lead to vaccine mismatch and larger epizootics. China initiated poultry vaccination against HPAI H7 in 2017. Although the prevalence of H5/H7 AIV in Chinese poultry was reduced by mass vaccination (*26*, *28*), stronger immune selection may increase the rate of viral adaptation (*67*, *68*). Vaccine-driven evolution may also increase infection risk for immunologically naive individuals, such as unvaccinated poultry and wild birds (*66*). Continued molecular epidemiological surveillance in vaccinated poultry and at the wild bird–poultry interface is vital for monitoring virus adaptive evolution and characterizing viral mutations (*69*, *70*).

Our study has several limitations. First, although we obtained robust results supported by different datasets, the heterogeneous sampling rates of infected wild birds and poultry may bias ancestral reconstructions. It is challenging to accurately identify ancestral host states in AIV lineages for countries with limited AIV surveillance in wild bird populations. Second, extracting viral lineages that circulate exclusively in vaccinated poultry was not possible due to the unknown vaccination status of the sampled hosts. Vaccination coverage can vary among different poultry species, even within the same country, and may exhibit heterogeneity among regions and over time. Third, the mixed distribution of various poultry species and the heterogeneity in sampling intensity across different regions complicate the inference of the specific impact of each poultry species on viral evolution and transmission. Fourth, the uncertainty and absence of viral genetic and surveillance data from many countries, both with and without vaccination, make it difficult at present to undertake a more quantitative comparative analysis of the association between vaccination levels and virus evolutionary dynamics. Last, although amino acid changes at certain positively selected sites on the HA gene were observed during the transition from the Chinese poultry lineage to the late-wild bird lineage, future experiments should be conducted to determine whether these mutations affect the transmissibility of H5 AIV in poultry and wild birds.

In conclusion, we find that vaccination in Asian poultry likely reduced the interspecies transmission of these viruses. We observe elevated rates of HA molecular evolution and adaptation in H5 AIV in Chinese poultry after the introduction of vaccination. Such circumstances may have increased the probability of infection in wild species at the wild bird–domestic bird interface and, in turn, altered the selective pressure acting upon the virus. As avian influenza continues to pose considerable challenges to wild and domestic animals, our research can help inform the development of preventive measures against AIV, such as global vaccination policies.

## MATERIALS AND METHODS

### Sequence data

We collated 12,818 publicly available HA gene sequences of H5 AIV, with a minimum length of 1200 nucleotides, sampled in Asia and Europe from January 1996 to February 2023 from the Global Initiative on Sharing All Influenza Data (GISAID). Only sequences with available information on date of collection, host species, and sampling location were retained for further analysis. We aligned the sequences using MAFFT v7.487 (*71*) with the autoselect algorithm (*72*). Recombinant sequences were detected using RDP4 package (*73*). Seven methods from the RDP package were used—RDP, GENECONV, Bootscan, Maxchi, Chimaera, SiScan, and 3seq—with default parameters, allowing a maximum acceptable *P* value of 0.05. A sequence was considered a potential recombinant if at least three of the seven detection methods showed a statistically significant difference (*P* < 0.05) (*74*, *75*). Using this criterion, 13 recombinant sequences were identified and removed. The maximum likelihood tree was estimated using FastTree v2.1.11 (*76*) under the automatically determined best-fit substitution model, followed by a root-to-tip regression analysis performed in TempEst v1.5.3 (*77*). We manually identified and removed 57 sequences with unexpectedly high or low levels of genetic divergence, given their sampling time. Last, 10,225 sequences were retained for further analysis.

On the basis of the sustained sampling efforts and the appropriate viral sample size (>500 sequences; fig. S1), six Asian countries (China, Bangladesh, Japan, Korea, Indonesia, and Vietnam) were selected. All European countries were collectively treated as a single group. To focus on the viral lineages that circulate in wild birds and poultry, the sequences were categorized into eight groups based on both geographic information and host type. These groups include wild birds (3118 sequences), European poultry (1939 sequences, without vaccination against H5 AIV), Japanese poultry (521 sequences, without vaccination against H5 AIV), Korean poultry (494 sequences, without vaccination against H5 AIV), Bangladeshi poultry (506 sequences, with vaccination against H5 AIV), Indonesian poultry (787 sequences, with vaccination against H5 AIV), Vietnamese poultry (1329 sequences, with vaccination against H5 AIV), and Chinese poultry (1531 sequences, with vaccination against H5 AIV).

We then downsampled these datasets in a stratified manner to create a more equitable distribution of AIV sequences between wild birds and poultry (main dataset): (i) wild bird dataset: randomly selected at most two sequences per month per country outside China and per month per province in China, comprising 1087 gene sequences from January 1999 to January 2023; (ii) European poultry dataset: randomly selected at most one sequence per month per country, including 338 gene sequences from January 1997 to January 2023; (iii) Japanese poultry dataset: randomly selected at most two sequences per month, including 72 HA gene sequences from January 2000 to January 2023; (iv) Korean poultry dataset: randomly selected at most two sequences per month, including 72 gene sequences from October 2008 to October 2022; (v) Bangladeshi poultry dataset: randomly selected at most one sequence per month, including 106 HA gene sequences from January 2007 to August 2022; (vi) Indonesian poultry dataset: randomly selected at most one sequence per month, including 121 HA gene sequences from January 2003 to March 2022; (vii) Vietnamese poultry dataset: randomly selected at most one sequence per month, including 151 HA gene sequences from 2003 to December 2021; and (viii) Chinese poultry dataset: randomly selected at most one sequence per month per province, including 462 gene sequences from January 1996 to March 2022.

As the ancestral reconstruction of discrete traits depends on sample availability, over- or undersampling of sequences from a particular trait can substantially affect the estimated ancestral states (*78*, *79*). Two different subsampling strategies were used to test the sensitivity of the model to sampling bias: (i) random sampling to reduced biased sampling between periods and groups and (ii) random sampling for an equivalent representation of different groups. Further details on the subsampling procedure and dataset composition can be found in the Supplementary Materials.

Considering that substantial mutations have also accumulated in the PB2 gene (*80*, *81*), we used a similar method to collate PB2 gene sequences from H5 AIV sampled from Asia and Europe for sensitivity analysis (see the Supplementary Materials).

### Phylogenetic inference

Evolutionary histories were estimated with the Bayesian phylogenetic package BEAST v1.10.4 (*82*), using the BEAGLE (*83*) library to improve computational speed. Specifically, we used an SRD06 substitution model (*42*), an uncorrelated lognormal relaxed clock (*42*), and a Gaussian Markov random field (GMRF) Bayesian Skygrid coalescent model (*84*). We subsequently used an eight-state discrete trait analysis (DTA) implemented in BEAST 1.10.4 to infer ancestral node hosts on empirical distributions of 500 time-calibrated trees sampled from the posterior tree distributions (*85*). An asymmetric model was used for the host discrete trait, which allows different rates of lineage movement between each pair of host states (*86*). Three independent Markov chain Monte Carlo (MCMC) runs were performed for 400 million steps and logged every 20,000 steps. The first 10% of each chain was discarded as burn-in. We confirmed the convergence of all chains in Tracer v1.7.1 (*87*), ensuring that the effective sample size (ESS) was > 200 for all parameters. A maximum clade credibility tree was estimated using TreeAnnotator v1.10.4 and subsequently visualized using FigTree v1.4.4 (http://tree.bio.ed.ac.uk/software/figtree) along with the R package ggtree v2.4.1 (*88*). The transition histories between host discrete states were summarized using the TreeMarkovJumpHistoryAnalyzer tool in BEAST, which extracts Markov jumps and their timings from a posterior tree distribution annotated with Markov jump histories (*89*). We used the BaTS 2.0 software (*90*) to investigate the uncertainty arising from phylogenetic error (grouped by sampling location and host), which was compared to a null hypothesis that there was no association between the phylogenetic structure and traits by performing tip randomization with 1000 replicates (table S11).

### Evolutionary analysis within host-specific populations

To infer virus dissemination within host-specific populations, we removed AIV phylogenetic clades that were determined to represent transmissions between different species (*43*). We extracted sequences for each host-specific lineage based on the aforementioned DTA and independently estimated the temporal phylogenies using the same substitution, clock, and tree models as described above. A strong phylogenetic temporal structure was observed in all host-specific lineages (fig. S16).

### Divergence analysis

To estimate site-specific synonymous and nonsynonymous substitutions in the different host-specific lineages, we applied a renaissance counting (*37*) procedure implementing a codon position–specific HKY nucleotide substitution model along with an uncorrelated lognormal relaxed clock (*42*) and the GMRF Bayesian Skygrid coalescent model (*84*). Sites under positive selection were also identified using complementary approaches. Specifically, FUBAR (Fast Unconstrained Bayesian AppRoximation) (*91*), SLAC (Single Likelihood Ancestor Counting) methods (*92*), and FEL (Fixed Effects Likelihood) methods (*92*) as implemented in Hyphy v2.5 were used (*93*).

The synonymous and nonsynonymous divergence for each host-specific lineage were calculated as the average Hamming distance between each sequenced isolate in that lineage and a reference sequence (NCBI Reference Sequence: AF144305). The estimated divergence was calculated by dividing the total number of observed differences between the isolate and reference nucleotide sequence that resulted in a substitution(nonsynonymous or synonymous) by the number of possible nucleotide mutations that could result in a substitution, weighted by kappa (*94*), the transition/transversion rate ratio, which was inferred from host-specific analysis using BEAST.

### Time series analysis

The extended CCM was applied to detect the time lags between the annual number of Markov jumps from Chinese poultry to wild birds (CPtoWB) and from wild birds to European poultry (WBtoEP) during 1996 to 2019 (*95*). First, we randomly selected 20 inferred Markov jump states among host populations from the converged MCMC chains. These 20 separate time series of Markov jumps were concatenated into the long-term chain for the extended CCM analysis. Then, we determined the optimal embedding dimension, which is optimal or very close to optimal for both variables. Last, the optimal value of the cross-mapping lag was determined by testing different values. The optimal cross-mapping lags are negative and with the magnitude of the lag roughly equal to the time delay between two time series (*95*). This analysis was performed using the R package rEDM v1.14.0 (*95*).

### Estimation of rates of adaptive substitution

We used an established population genetic method related to the McDonald-Kreitman test (*36*, *68*) to estimate the number of adaptive substitutions per codon per year in the HA H5 gene from the Chinese poultry lineage. We used a consensus of HA sequences from the earliest time point (sampled in 1996) as an outgroup to estimate ancestral and derived site frequencies at subsequent time points. A bootstrap analysis with 1000 replicates was performed to assess statistical uncertainty.

## Supplementary Materials

### This PDF file includes:

Supplementary Information Text Figs. S1 to S16 Tables S1 to S11 References
