# Supplementary material for: Association of poultry vaccination with interspecies transmission and molecular evolution of H5 subtype avian influenza virus
Source: Sci Adv. 2025 Jan 22;11(4):eado9140. doi: 10.1126/sciadv.ado9140 (PMC11753422; doi:10.1126/sciadv.ado9140)
Supplement: Supplementary file 1 — Supplementary Information Text Figs. S1 to S16 Tables S1 to S11 References [file sciadv.ado9140_sm.pdf]

Science Advances
AAAS

Supplementary Materials for

Association of poultry vaccination with interspecies transmission and molecular evolution of H5 subtype avian influenza virus

Bingying Li *et al.*

Corresponding author: Huaiyu Tian, tianhuaiyu@gmail.com; Oliver G. Pybus, opybus@rvc.ac.uk

Sci. Adv. **11**, eado9140 (2025)
DOI: 10.1126/sciadv.ado9140

This PDF file includes:

Supplementary Information Text
Figs. S1 to S16
Tables S1 to S11
References

Supplementary Information Text

Sampling strategies for sensitivity analysis of interspecies transmission between wild birds, vaccinated poultry and unvaccinated poultry

We performed multiple random downsamples on the original datasets in a stratified manner to obtain more robust historical dynamics of interspecies transmission among populations:

1. random sampling to reduced biased sampling between periods and groups:

Main dataset: reduce sampling bias by increasing sampling intensity in wild bird populations and in poultry populations where sampling intensity is lower.

(1) wild bird dataset: 1087 gene sequences, randomly selected at most 2 sequences per month per country outside China and per month per province in China.

(2) poultry dataset: 1322 gene sequences, randomly selecting at most 1 sequence per month per country (randomly selecting at most 2 sequences per month in Japan and Korea due to the insufficient sequences).

Dataset R1: reduce sampling bias by increasing sampling intensity in countries with low wild bird monitoring intensity.

(1) wild bird dataset: 793 gene sequences, randomly selected at most 2 sequences per month per country in Europe and per month per province in China, randomly selecting at most 2 sequences per month in Japan, Korea, Bangladesh, Indonesia and Vietnam.

(2) poultry dataset: 1326 gene sequences, randomly selecting at most 1 sequence per month per country (randomly selecting at most 2 sequences per month in Japan and Korea due to the insufficient sequences).

Dataset R2: increase sampling intensity in countries with low wild bird monitoring intensity and standardize sampling intensity across poultry populations to reduce sampling bias.

(1) wild bird dataset: 795 gene sequences, randomly selected at most 2 sequences per month per country outside China and per month per province in China, randomly selecting at most 2 sequences per month in Japan, Korea, Bangladesh, Indonesia and Vietnam.

(2) poultry dataset: 1266 gene sequences, randomly selecting at most 1 sequence per month per country.

2. random sampling for an equivalent representation of different groups:

Dataset R3: ensure similar sample size in wild birds, vaccinated poultry and unvaccinated poultry populations.

(1) wild bird dataset: 553 gene sequences, randomly selected at most 4 sequence per month from Europe, China, Japan, Korean, Bangladesh, Indonesia and Vietnam, respectively.

(2) vaccinated poultry: 537 gene sequences, randomly selected at most 1 sequence per month from China, Bangladesh, Indonesia and Vietnam, respectively.

(3) unvaccinated poultry: 481 gene sequences, randomly selected at most 1 sequence per month per country in Europe, and randomly selected at most 2

sequence per month from Japan and Korea.

Dataset R1: 1,326 H5 AIV haemagglutinin (HA) gene sequences of domestic poultry and 793 sequences of wild birds. We performed random downsampling of the datasets, ensuring equal sampling frequency for wild birds and poultry sequences: 1) wild birds dataset (randomly selected at most 1 sequence per month per country in Europe and per month per province in China, and randomly selected at most 2 sequences per month per country among Japan, Korean, Bangladesh, Indonesia and Vietnam), comprising 793 HA gene sequences from January 1999 to January 2023; 2) European poultry dataset (randomly selected at most 1 sequence per month per country), including 338 HA gene sequences from January 1997 to January 2023; 3) Japanese poultry dataset (randomly selected at most 2 sequences per month), including 76 HA gene sequences from January 2000 to January 2023; 4) Korean poultry dataset (randomly selected at most 2 sequences per month), including 74 gene sequences from October 2008 to October 2022; 5) Bangladeshi poultry dataset (randomly selected at most 1 sequence per month), including 106 HA gene sequences from May 2007 to August 2022. 6) Indonesian poultry dataset (randomly selected at most 1 sequence per month), including 121 HA gene sequences from January 2003 to March 2022. 7) Vietnamese poultry dataset (randomly selected at most 1 sequence per month), including 151 HA gene sequences from 2003 to December 2021. 8) Chinese poultry dataset (randomly selected at most 1 sequence per month per province), including 460 HA gene sequences from January 1996 to March 2022.

Dataset R2: 1266 H5 AIV haemagglutinin (HA) gene sequences of domestic poultry and 795 sequences of wild birds. We performed random downsampling of the datasets, ensuring equal sampling frequency for wild birds and poultry sequences: 1) wild birds dataset (randomly selected at most 1 sequence per month per country in Europe and per month per province in China, and randomly selected at most 2 sequences per month per country among Japan, Korean, Bangladesh, Indonesia and Vietnam), comprising 795 HA gene sequences from January 1999 to January 2023; 2) European poultry dataset (randomly selected at most 1 sequence per month per country), including 338 HA gene sequences from January 1997 to January 2023; 3) Japanese poultry dataset (randomly selected at most 1 sequence per month), including 47 HA gene sequences from January 2000 to January 2023; 4) Korean poultry dataset (randomly selected at most 1 sequence per month), including 42 gene sequences from October 2008 to October 2022; 5) Bangladeshi poultry dataset (randomly selected at most 1 sequence per month), including 106 HA gene sequences from May 2007 to August 2022. 6) Indonesian poultry dataset (randomly selected at most 1 sequence per month), including 121 HA gene sequences from January 2003 to March 2022. 7) Vietnamese poultry dataset (randomly selected at most 1 sequence per month), including 151 HA gene sequences from 2003 to December 2021. 8) Chinese poultry

dataset (randomly selected at most 1 sequence per month), including 460 HA gene sequences from January 1996 to March 2022.

Dataset R3: 1018 H5 AIV haemagglutinin (HA) gene sequences of domestic poultry and 553 sequences of wild birds. We performed random downsampling of the datasets, ensuring equal sampling size for wild birds and poultry sequences: 1) wild birds dataset (randomly selected at most 4 sequence per month from Europe, China, Japan, Korean, Bangladesh, Indonesia and Vietnam, respectively), comprising 553 HA gene sequences from January 1996 to January 2023; 2) vaccinated poultry dataset (randomly selected at most 1 sequence per month from China, Bangladesh, Indonesia and Vietnam, respectively), including 537 HA gene sequences from January 1997 to August 2022; 3) unvaccinated poultry dataset (randomly selected at most 1 sequence per month per country in Europe, and randomly selected at most 2 sequence per month from Japan and Korea), including 481 HA gene sequences from January 1997 to January 2023.

Sampling strategies for sensitivity analysis of time lags in interspecies transmission between Chinese poultry, wild birds and European poultry

The time lag in virus transmission was observed from Chinese poultry to wild birds, and then from wild birds to European poultry. To verify the stability of this transmission pattern, we performed sensitivity analyzes using diverse datasets. Given the singular transmission chain involving Chinese poultry, wild birds, and European poultry, the sensitivity analyses were exclusively based on virus sequences from these specific sources.

Dataset S1: 800 H5 AIV haemagglutinin (HA) gene sequences of domestic poultry, 850 of wild birds and 148 of environment samples. As human and other mammals are considered as almost always terminal hosts of AIVs, we only combined the sequences sampled from the environment with the main dataset (see Method section in the main text) to obtain the relatively complete phylogenies and interspecies transmission of the virus: 1) wild birds dataset (randomly selected at most 2 sequences per month per country in Europe and per month per province in China), including 850 HA gene sequences from January 1999 to January 2023; 2) European poultry dataset (randomly selected at most 1 sequence per month per country), including 338 HA gene sequences from January 1997 to January 2023; 3) Chinese poultry dataset (randomly selected at most 1 sequence per month per province), including 462 HA gene sequences from January 1996 to March 2022; 4) Environment dataset (randomly selected at most 1 sequence per month per country in Europe and province in China), including 148 HA gene sequences from January 2005 to March 2023.

Dataset S2: 800 H5 AIV haemagglutinin (HA) gene sequences of domestic poultry and 531 sequences of wild birds. We performed random downsampling of the

datasets, ensuring equal sampling frequency for wild birds, Chinese poultry, and European poultry sequences: 1) wild birds dataset (randomly selected at most 1 sequence per month per country in Europe and per month per province in China), including 531 HA gene sequences from January 1999 to January 2023; 2) European poultry dataset (randomly selected at most 1 sequence per month per country), including 338 HA gene sequences from January 1997 to January 2023; 3) Chinese poultry dataset (randomly selected at most 1 sequence per month per province), including 462 HA gene sequences from January 1996 to March 2022.

Dataset S3: 800 H5 AIV haemagglutinin (HA) gene sequences of domestic poultry and 850 of wild birds. To mitigate potential sampling biases, we randomly subsampled the datasets using the same sampling strategy that was applied to the main dataset (see Method section in main text), and obtained a dataset with roughly equal numbers of sequences from wild bird and poultry: 1) wild birds dataset (randomly selected at most 2 sequences per month per country in Europe and per month per province in China), including 850 HA gene sequences from January 1999 to January 2023; 2) European poultry dataset (randomly selected at most 1 sequence per month per country), including 338 HA gene sequences from January 1997 to January 2023; 3) Chinese poultry dataset (randomly selected at most 1 sequence per month per province), including 462 HA gene sequences from January 1996 to March 2022.

Sampling strategies for sensitivity analysis of evolutionary rate in vaccinated poultry

Chinese poultry has relatively abundant and complete virus sequence samples, so we calculated the evolutionary rate of H5 AIV PB2 gene in wild birds and Chinese poultry.

Dataset PB2: 729 H5 AIV PB2 gene sequences of domestic poultry and 817 of wild birds. To mitigate potential sampling biases, we randomly subsampled the datasets using the same sampling strategy that was applied to the main dataset (see Method section in main text), and obtained a dataset with roughly equal numbers of sequences from wild birds and poultry: 1) wild birds dataset (randomly selected at most 2 sequences per month per country in Europe and per month per province in China), including 817 HA gene sequences from April 1996 to May 2023; 2) European poultry dataset (randomly selected at most 1 sequence per month per country), including 327 HA gene sequences from January 1997 to May 2023; 3) Chinese poultry dataset (randomly selected at most 1 sequence per month per province), including 402 HA gene sequences from January 1997 to March 2022.

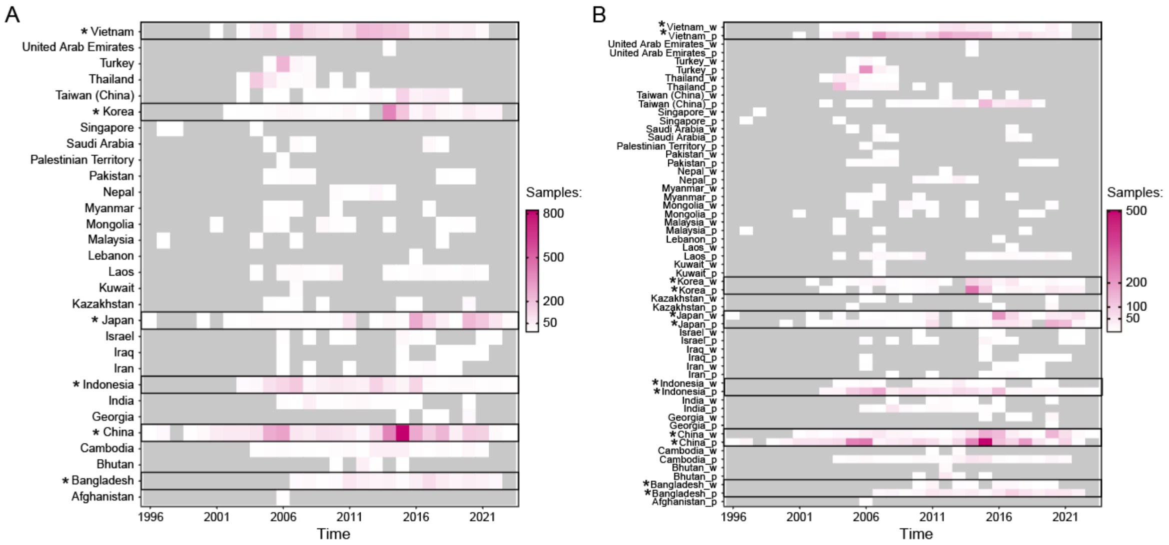

Fig S1. Number of H5 AIV HA gene sequences sampled in Asian countries and regions. (A) Total sample size. (B) Number of gene sequences sampled from different hosts (w: sampled from wild birds, p: sampled from poultry). Countries with continuous sampling and an adequate sample size (>500 sequences), from which data were retained for subsequent analysis, are marked with a black border and an asterisk.

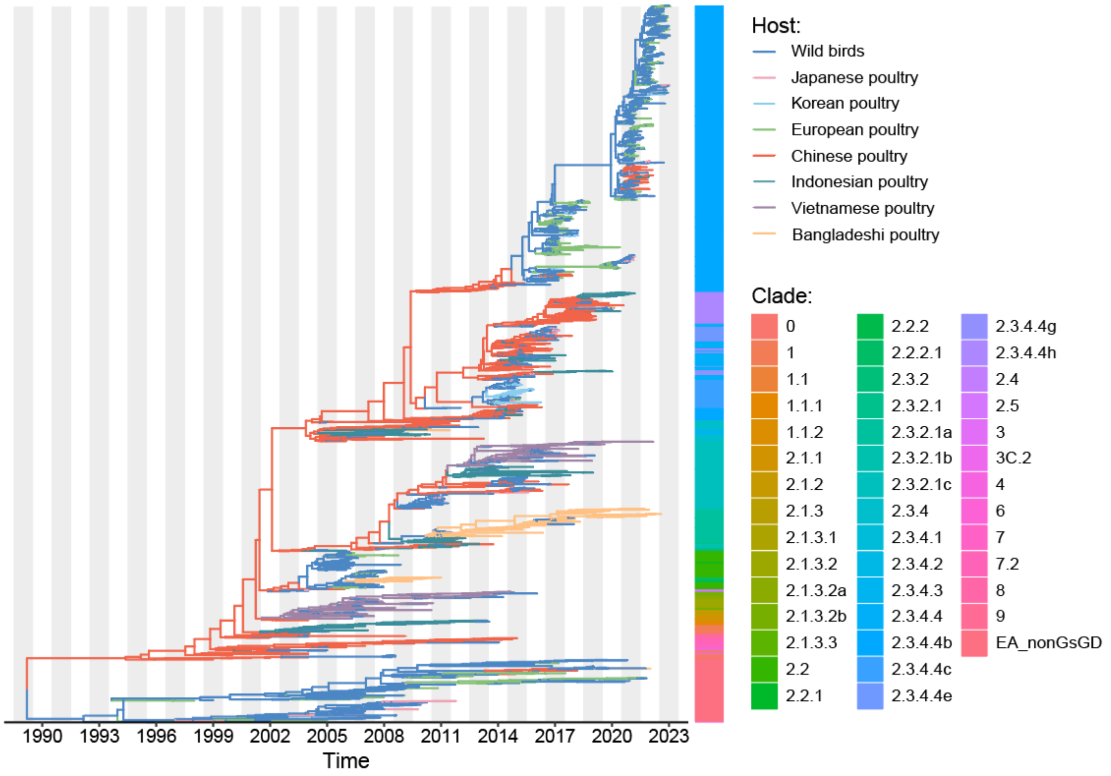

Fig S2. The maximum clade credibility tree of H5 AIV HA gene sequences sampled from 1996 to 2023. Tree tips are coloured according to the host from which the sequence was sampled, while internal branches represent ancestral host states inferred using the asymmetric discrete phylogenetic model (dark blue: wild birds; orange: Chinese poultry; light green: European poultry; purple: Indonesian poultry; pink: Japanese poultry; light blue: Korean poultry; dark green: Vietnamese poultry; yellow: Bangladeshi poultry). The lineage information of each tip is provided by GISAID.

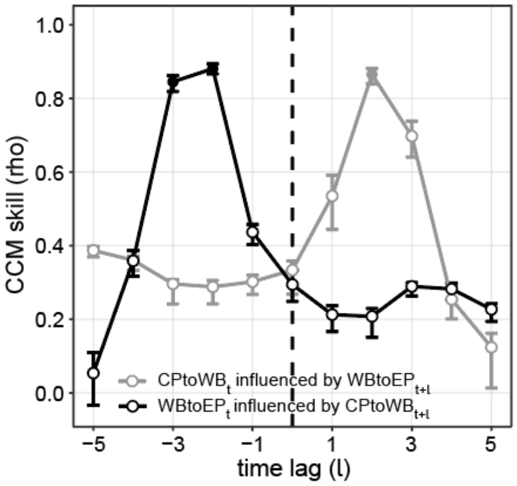

Fig S3. Extended convergent cross mapping (CCM) analysis of interspecies transmission. CPtoWB$_t$: the mean Markov jumps from Chinese poultry to wild bird in year t; WBtoEP$_t$: the mean Markov jumps from wild bird to European poultry in year t. We randomly selected 20 states from the converged MCMC chains and combined them into an extended chain for CCM analysis. Black line: using CPtoWB$_{t+l}$ to predict WBtoEP$_t$. The negative optimal CCM skill indicated that CPtoWB can effectively predict WBtoEP 2-3 years ahead. Gray line: using WBtoEP$_{t+l}$ to predict CPtoWB$_t$. Conversely, the positive optimal CCM skill indicated that WBtoEP can predict CPtoWB 2-3 years in the past, but implies that forecasting future CPtoWB from WBtoEP is not feasible.

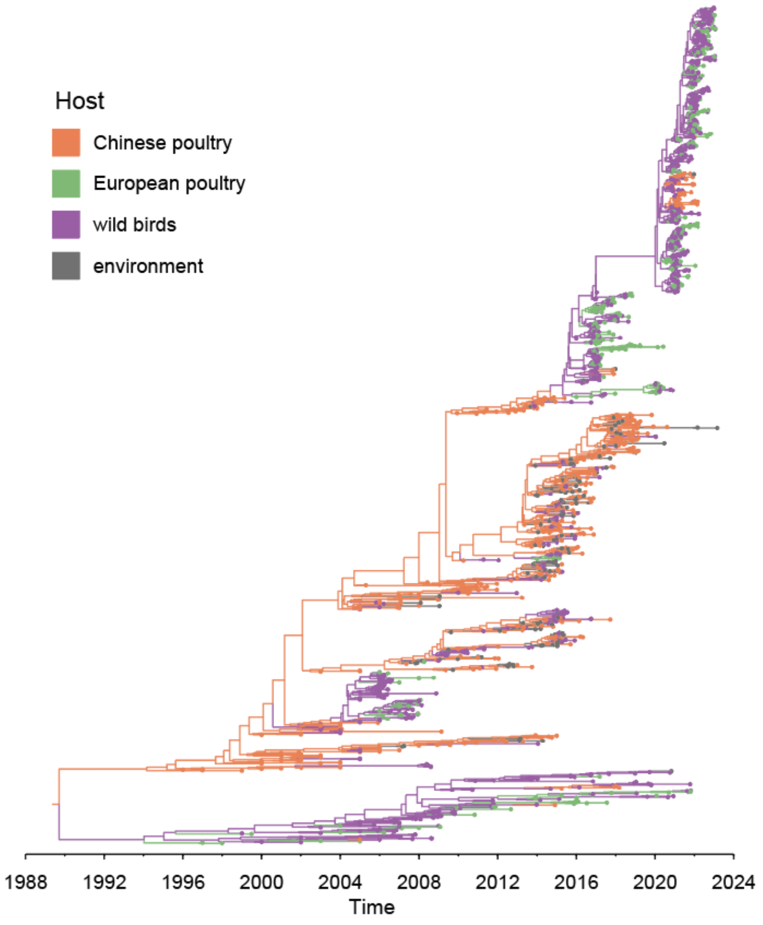

Fig S4. The maximum clade credibility tree of H5 AIV HA gene sampled from 1996 to 2023. The tips are colored based on their host states, while internal branches are colored according to ancestral states inferred using the asymmetric discrete phylogenetic model with Bayesian Stochastic Search Variable Selection (purple: wild birds; orange: Chinese poultry; light purple: European poultry; gray: environment). The phylogeny is inferred from *Dataset S1*.

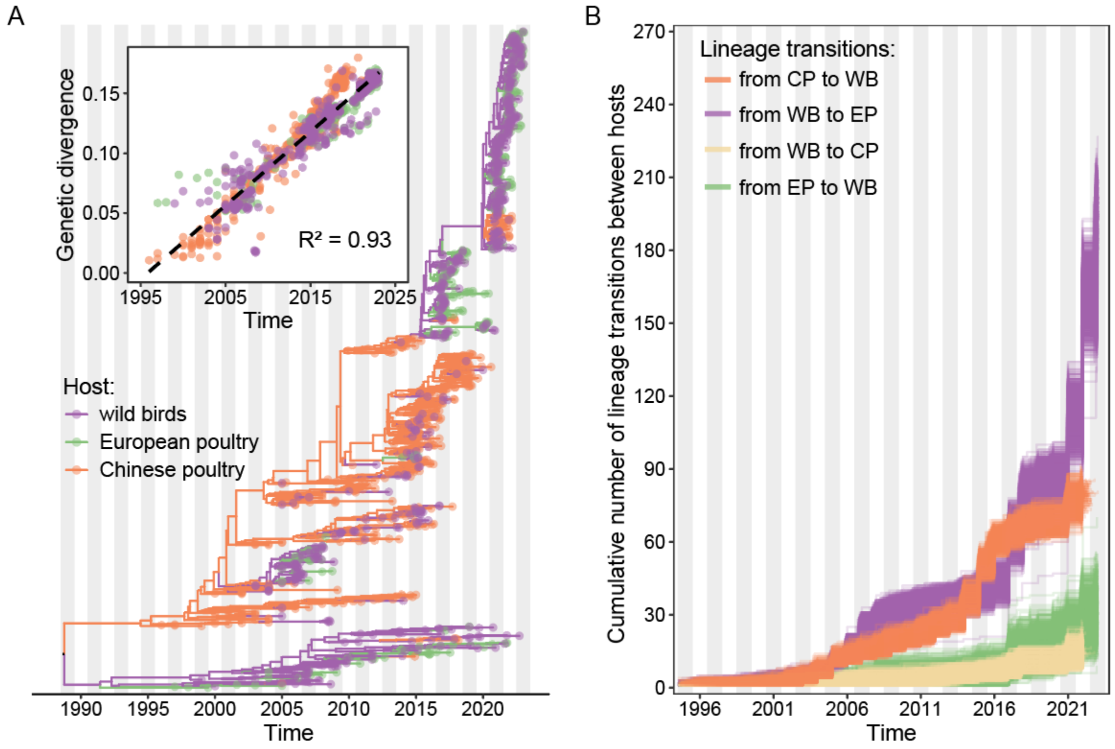

Fig S5. Dynamic lineage transmissions of H5 AIV HA gene among three host populations inferred from Dataset S2. (A) The maximum clade credibility tree of H5 AIV HA gene sampled from 1996 to 2022. The tips are colored based on their host states, while internal branches are colored according to ancestral states inferred using the asymmetric discrete phylogenetic model with Bayesian Stochastic Search Variable Selection (purple: wild birds; orange: Chinese poultry; light purple: European poultry). Inset: a root-to-tip regression of genetic divergence against the dates of sample collection. (B) The cumulative lineage transitions of the HA gene between the three host states were summarized from the posterior samples of an asymmetric discrete phylogenetic analysis.

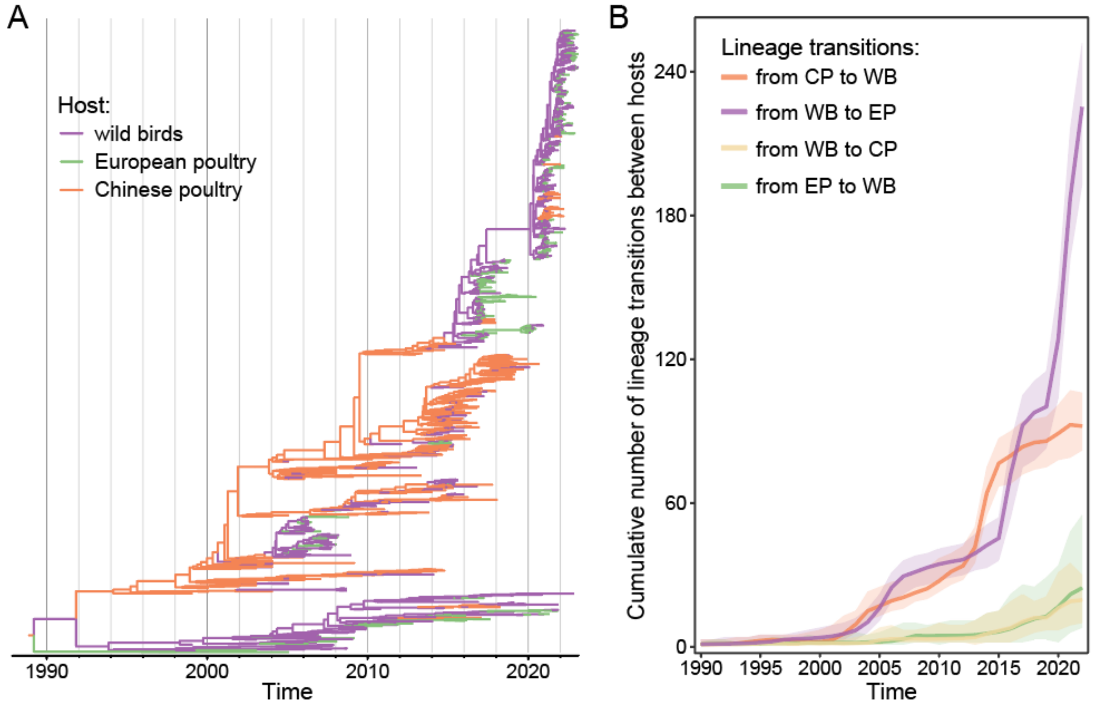

Fig S6. Dynamic lineage transmissions of H5 AIV HA gene among three host populations inferred from Dataset S3. (A) The maximum clade credibility tree of H5 AIV HA gene sampled from 1996 to 2022. The tips are colored based on their host states, while internal branches are colored according to ancestral states inferred using the asymmetric discrete phylogenetic model with Bayesian Stochastic Search Variable Selection (purple: wild birds; orange: Chinese poultry; light purple: European poultry). (B) The cumulative lineage transitions of the HA gene between the three host states were summarized from the posterior samples of an asymmetric discrete phylogenetic analysis. The solid coloured lines represent the annual mean values of the cumulative lineage transitions and the shaded areas show the 95% highest posterior density credible intervals of that estimate.

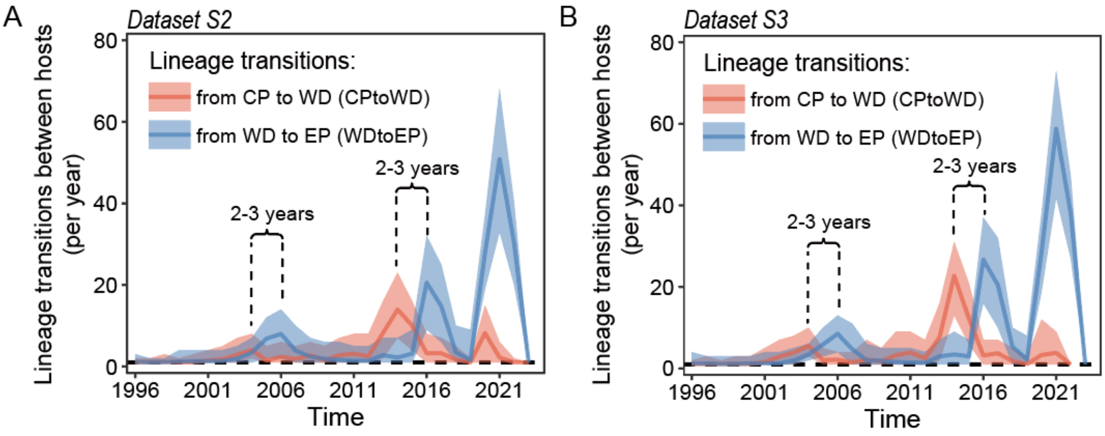

Fig S7. Time series of the annual mean lineage transitions of H5 AIV HA gene between wild birds (WB), Chinese poultry (CP) and European poultry (EP). Panel (A) displays results derived from Dataset S2, while Panel (B) derived from Dataset S3.

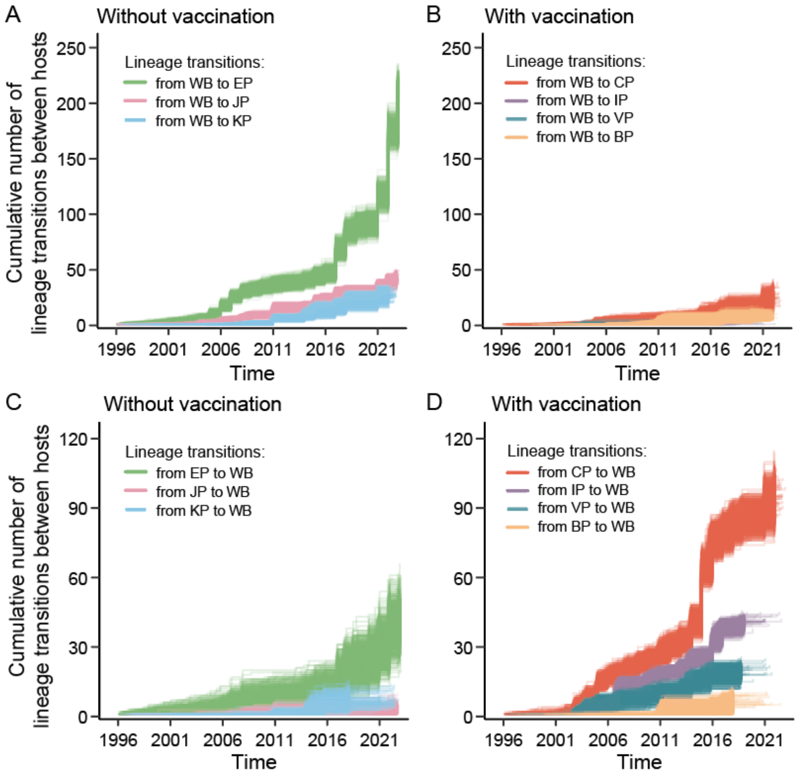

Fig S8. Inter-species lineage transmission between wild birds and poultry populations with different vaccination statues. (**A**) Accumulation of lineage transitions from wild birds to unvaccinated poultry populations. (**B**) Accumulation of lineage transitions from wild birds to vaccinated poultry populations. (**C**) Accumulation of lineage transitions from unvaccinated poultry populations to wild birds. (**D**) Accumulation of lineage transitions from vaccinated poultry populations to wild birds. The plots were summarized from a posterior sample of trees from the asymmetric discrete phylogenetic model (WB: wild birds; CP: Chinese poultry; EP: European poultry, IP: Indonesian poultry; JP: Japanese poultry; KP: Korean poultry; VP: Vietnamese poultry; BP: Bangladeshi poultry).

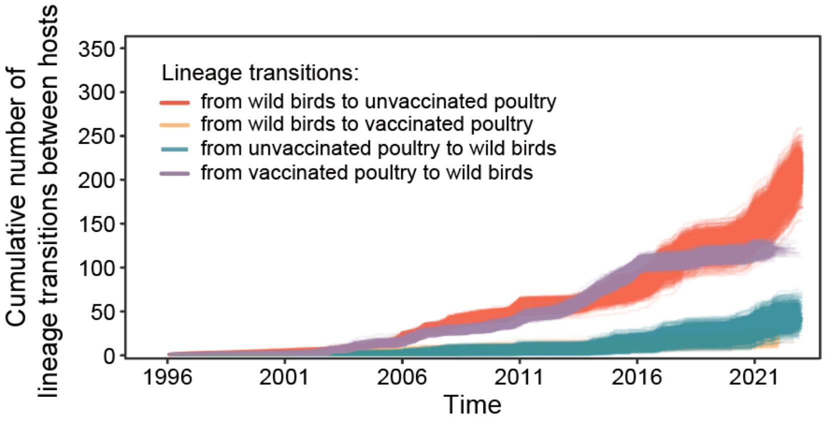

Fig S9. Inter-species lineage transmission between wild birds and poultry populations with different vaccination statues inferred from Dataset R3.

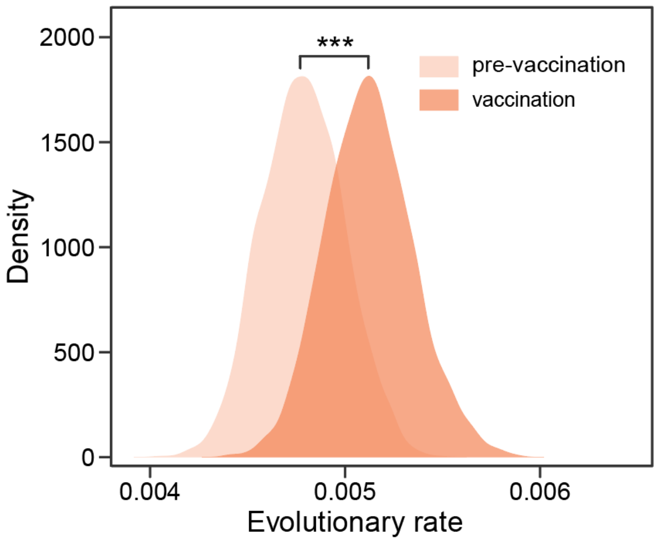

Fig S10. Evolutionary rate (subs/site/year) of the Chinese poultry lineage in pre-vaccination era and vaccination era. A time-dependent rate (TDR) model was implemented.

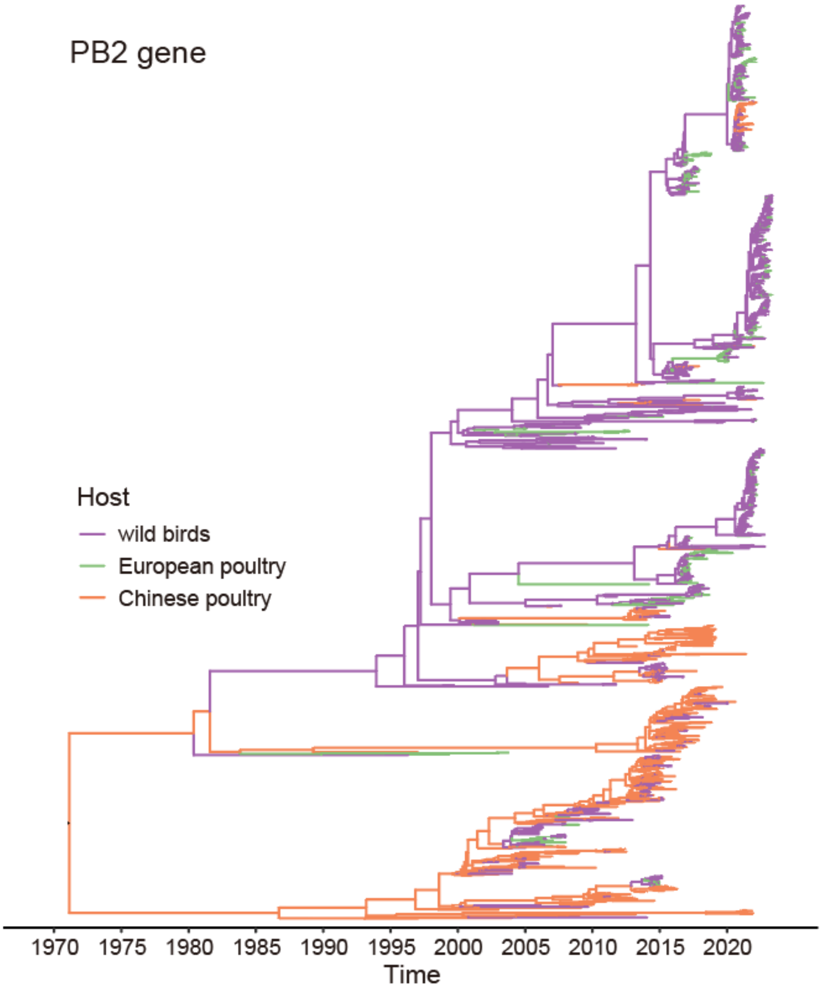

Fig S11. Maximum clade credibility tree of the PB2 gene of H5 AIV sampled from 1996 to 2023 in China and Europe. The tips are colored based on their host states (purple: wild birds; orange: Chinese poultry; green: European poultry), while internal branches are colored according to ancestral states inferred using the asymmetric discrete phylogenetic model with Bayesian Stochastic Search Variable Selection (purple: wild birds; orange: Chinese poultry; green: European poultry).

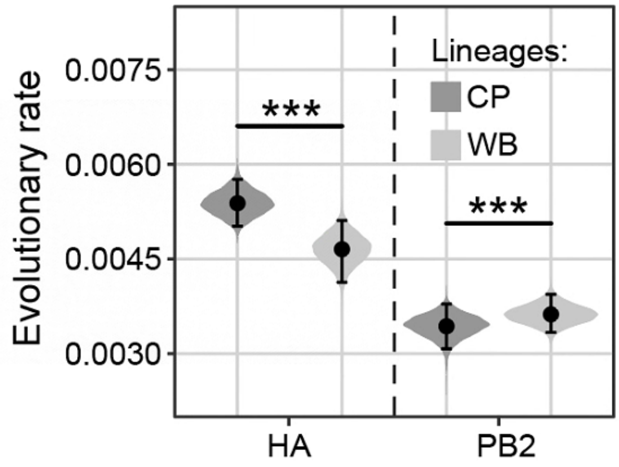

Fig S12. The estimated substitution rates (subs/site/year) of HA gene and PB2 gene of Chinese poultry and wild bird lineages. For the PB2 gene, there are two host-specific lineages: the Chinese poultry lineage (CP) and the wild bird lineage (WB). The lineages of HA gene were categorized into two lineages: the Chinese poultry lineage (CP), the wild bird lineage (WB, consisting of early-wild bird lineage and late-wild bird lineage). The accompanying dot and the whisker plots indicate the mean and the 95% highest posterior density credible intervals for these estimates. We used Wilcoxon tests to assess differences in pairwise substitution rates between lineages, with statistical significance levels of $p < 0.05$ (*), $p < 0.01$ (**), and $p < 0.001$ (***) denoting statistically significant, highly significant, and extremely significant differences, respectively.

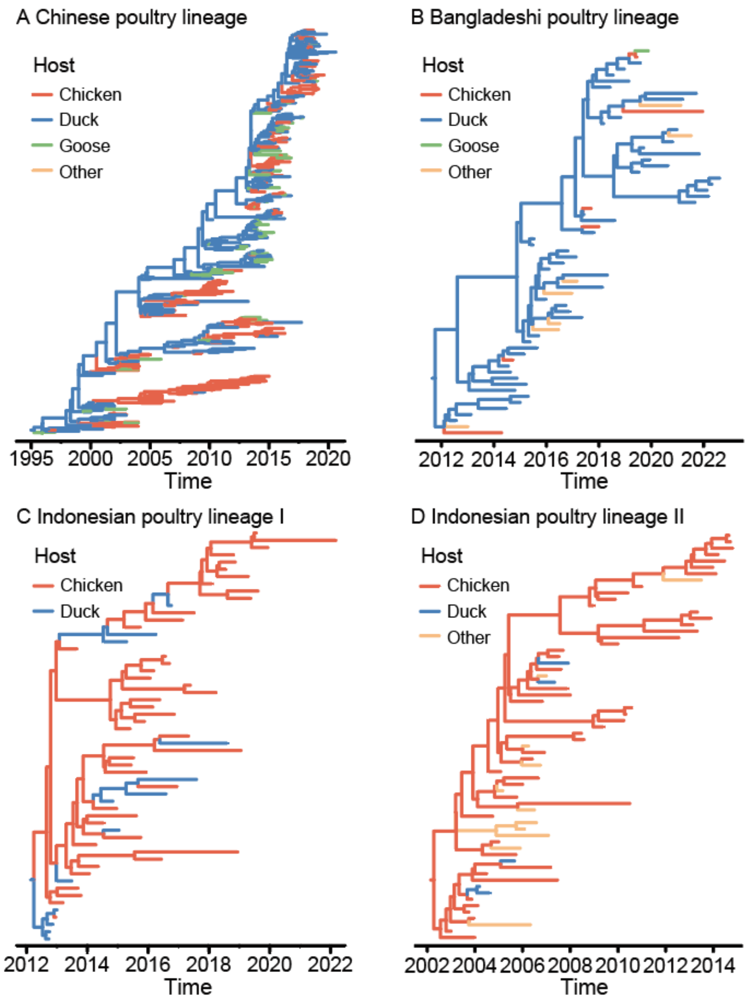

Fig S13. The maximum clade credibility trees of different host-specific lineages. (A) Chinese poultry lineage. (B) Bangladeshi poultry lineage. (C) Indonesian poultry lineage I. (D) Indonesian poultry lineage II. The tips are colored based on their host states, while internal branches are colored according to ancestral states inferred using the asymmetric discrete phylogenetic model with Bayesian Stochastic Search Variable Selection.

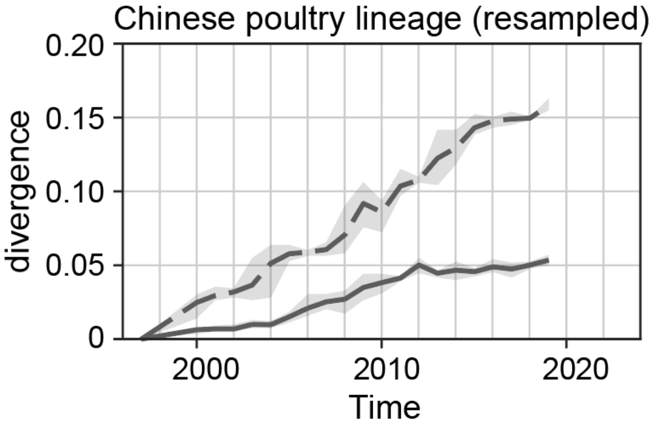

Fig S14. Nonsynonymous (solid lines) and synonymous (dashed lines) divergence of the HA gene of the Chinese poultry lineage through time. The Chinese poultry lineage was randomly resampled to include at most 3 sequences per year. Divergences were computed using 1-year sliding windows. Shaded region shows 95% confidence intervals.

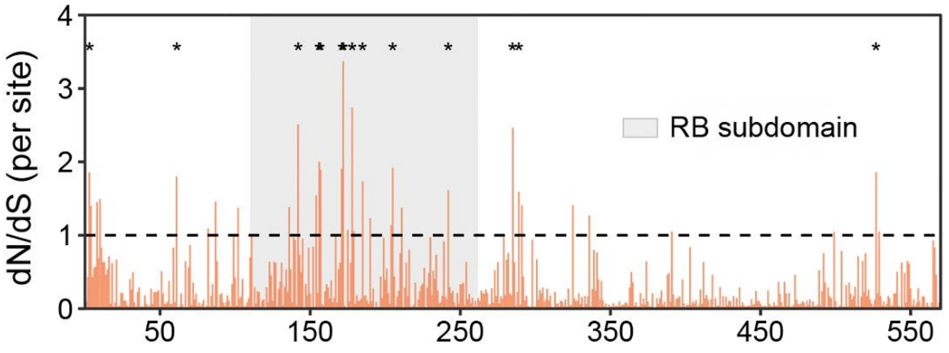

Fig S15. Mean nonsynonymous/synonymous divergence (dN/dS) of each site in H5 AIV HA gene of the Chinese poultry lineage. The sites identified as experiencing significant positive selection through the renaissance counting method are marked with an asterisk. The horizontal dashed line indicates the dN/dS ratio of 1, and the grey shaded regions indicate the receptor binding subdomain sites.

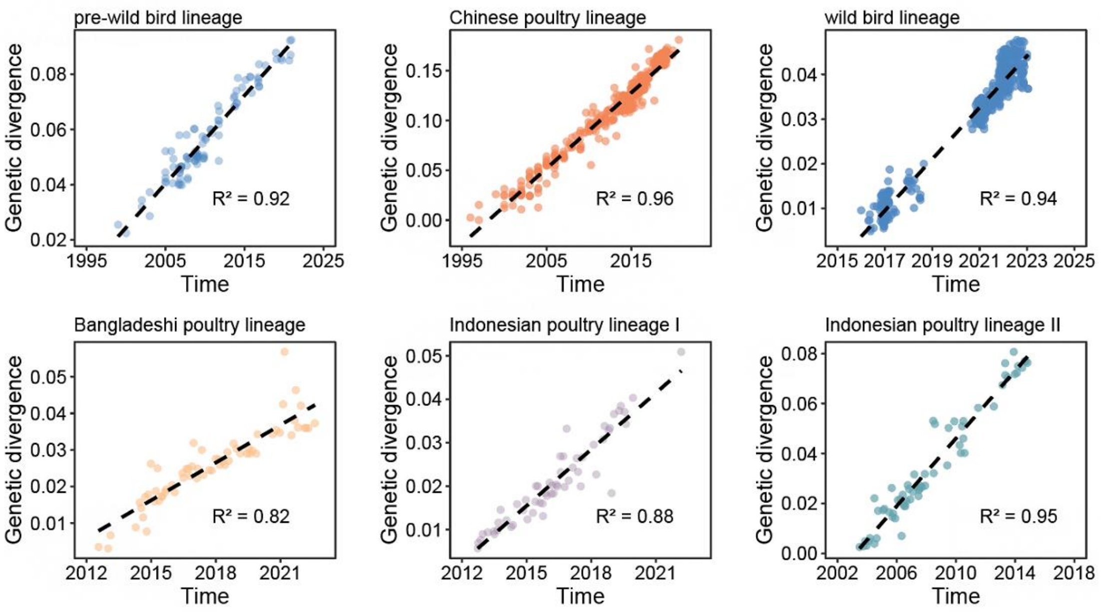

Fig S16. Temporal signal of genetic divergence for host-specific lineages.

Table S1. Avian influenza vaccines have been used to control the highly pathogenic H5 avian influenza.

Country	Seed virus	HA donor virus (subtype) (clade)[b]	Application period	Ref.
China [a]	inactivated N-28	A/turkey/England/N-28/73 (LP H5N2) (-)	2004-2006	(24)
	inactivated H5-Re1	GS/GD/1/1996 (H5N1) (0)	03/2004-3/2008	(97)
	inactivated H5-Re4	CK/SX/2/2006 (H5N1) (7.2)	07/2006-4/2014	
	inactivated H5-Re5	DK/AH/1/2006 (H5N1) (2.3.4)	03/2008-6/2012	
	inactivated H5-Re6	DK/GD/S1322/2010 (H5N1) (2.3.2)	06/2012-9/2017	
	inactivated H5-Re7	CK/LN/S4092/2011 (H5N1) (7.2)	04/2014-9/2017	
	inactivated H5-Re8	CK/GZ/4/2013 (H5N1) (2.3.4.4g)	12/2015-2/2018	
	inactivated H5-Re11	DK/GZ/S4184/2017 (H5N6) (2.3.4.4h)	12/2018-2/2021	
	inactivated H5-Re12	CK/LN/SD007/2017 (H5N1) (2.3.2.1f)	12/2018-2/2021	
	inactivated H5-Re13	DK/FJ/S1424/2020 (H5N6) (2.3.4.4h)	01/2022-	
	inactivated H5-Re14	WS/SX/4-1/2020 (H5N8) (2.3.4.4b)	01/2022-	
	live vector rFPV-HA-NA	GS/GD/1/1996(H5N1) (0)	01/2005-12/2005	(98)
	live vector rL-H5	GS/GD/1/1996(H5N1) (0)	01/2007-03/2008	
	live vector rL-H5-5	DK/AH/1/2006(H5N1) (2.3.4)	03/2008-06/2012	
	live vector rL-H5-6	DK/GD/S1322/2010(H5N1) (2.3.2)	06/2012-12/2015	
	live vector rL-H5-8	CK/GZ/4/2013(H5N1) (2.3.4.4g)	01/2016-	
Vietnam	inactivated H5-Re1 (H5N1)	GS/GD/1/1996 (H5N1) (clade 0)	2005-2009	(99, 100)
	inactivated H5-Re5 (H5N1)<br>inactivated H5-Re6 (H5N1)	DK/AH/1/2006 (H5N1) (clade 2.3.4)<br>DK/GD/S1322/2010 (H5N1) (clade 2.3.2)	2010-2014	
	inactivated H5-Re6 (H5N1)<br>inactivated H5-Re8 (H5N1)	DK/GD/S1322/2010 (H5N1) (clade 2.3.2)<br>CK/GZ/4/2013 (H5N1) (clade 2.3.4.4g)	2015-	
Bangladesh	Vectormune HVT-AI (Ceva)<br>inactivated Re-6 (Merial)<br>inactivated Nobilis H5 (MSD)	A/swan/Hungary/4999/2006 (H5N1) (clade 2.2)<br>A/dk/GD/S1322/2010 (H5N1) (clade 2.3.2.1)<br>A/duck/Potsdam/1402/1986 (LP H5N2) (-)	2012-2013	(23)
	inactivated Re-6 (Merial),<br>Vectormune HVT-AI (Ceva)	A/dk/GD/S1322/2010 (H5N1) (clade 2.3.2.1)<br>A/swan/Hungary/4999/2006 (H5N1) (clade 2.2)	2014-	
Indonesia [c]	inactivated vaccine	A/turkey/England/N28/1973 (LP H5N2) (-)<br>A/chicken/Legok/2003 (H5N1) (clade 2.1.1)<br>A/goose/GD/1/96 (H5N1) (clade 0)<br>A/turkey/Wisconsin/1968 (LP H5N9) (-)<br>A/chicken/Hidalgo/28159-232/1994 (LP H5N2) (-)<br>A/chicken/Vietnam/C57/2004 (LP H5N3) (-)<br>A/chicken/West Java/Pwt-Wij/2006 (H5N1)	2005-2015	(101, 102)

	(clade 2.1.3.2)<br>A/chicken/West Java/ 30/2007 (H5N1)<br>(clade 2.1.3.2)<br>A/chicken/Mexico/232/1994 (LP H5N2) (-)<br>A/duck/Potsdam/1402/1986 (LP H5N2) (-)

a China: Only vaccine seed viruses prepared by the Harbin Veterinary Research Institute are listed in this table. When two or three seed viruses are used at the same time in China, it means that these seed viruses are used for bivalent or trivalent inactivated vaccine production.

b Abbreviations: GS, goose; CK, chicken; DK, duck; WS, whooper swan; PG, pigeon; GD, Guangdong; SX, Shanxi; AH, Anhui; LN, Liaoning; GZ, Guizhou; FJ, Fujian; SH, Shanghai; GX, Guangxi; IM, Inner Mongolia; YN, Yunnan.

c The specifics of the vaccine strains (vaccine seeds) and their usage periods were missing.

Table S2. The intensity of surveillance for H5 AIV in poultry and wild bird populations by country/continent.

Continent	Country	Geographic area of the country for poultry (km^2) †	Geographic area of the country for wild birds (km^2) *	Number of sampled sequences from poultry	Number of sampled sequences from wild birds	Sample density in poultry (10E-3 sample/km^2)	Sample density in wild birds (10E-3 samples /km^2)
Asia	China	5645201	7322401	2461	865	0.44	0.12
	Bangladesh	136279	140978	579	80	4.25	0.57
	Indonesia	1326054	1805944	1043	97	0.79	0.05
	Japan	357709	315944	1641	318	4.59	1.01
	South Korea	91811	89589	599	551	6.52	6.15
	Vietnam	308488	330354	580	354	1.88	1.07
	Turkey	524188	775605	269	24	0.51	0.03
	Thailand	447077	511437	198	93	0.44	0.18
	Cambodia	115262	181704	243	15	2.11	0.08
Europe		7838437	15151968	2479	2843	0.32	0.19
Africa	Egypt	93248	943246	1532	56	16.43	0.06
	Nigeria	854497	883277	360	47	0.42	0.05
	South Africa	970308	1063563	111	98	0.11	0.09
North America	United States	1268631	8243521	1206	975	0.95	0.12
	Mexico	1098857	1931524	366	9	0.33	0.00
	Canada	326841	4975339	114	199	0.35	0.04

†Mean livestock density was calculated using data from the Gridded Livestock of the World (GLW) website, hosted by Food and Agriculture Organization (FAO; https://dataverse.harvard.edu/dataverse/glw). Regions with <10 birds per square kilometer were excluded from the calculation.

* Estimation based on wild bird distribution probability grid data (*103*). If the probability of a wild bird appearing in a grid is greater than 0.5, the grid is considered to belong to the wild bird's geographical distribution area. The total area of all grids with grid values greater than 0.5 can be calculated to obtain the geographical distribution area of wild birds in a certain country/region.

Table S3. The posterior support of each host branch in different datasets.

Dataset	Branch	Host. prob
main text	Chinese poultry section	0.9882
	Early-wild bird section	0.7902
	Late-wild bird section	0.9822
	Bangladeshi poultry lineage	1
	Indonesian poultry lineage I	0.9882
	Indonesian poultry lineage II	1
Dataset S1	Chinese poultry section	0.9947
	Early-wild bird section	0.824
	Late-wild bird section	0.9921
Dataset S2	Chinese poultry section	1
	Early-wild bird section	0.8275
	Late-wild bird section	0.9935
Dataset S3	Chinese poultry section	0.9987
	Early-wild bird section	0.8507
	Late-wild bird section	0.9934

Table S4. The result of null hypothesis tests.

Statistic	observed			null			significance
	mean	lower 95% CI	upper 95% CI	mean	lower 95% CI	upper 95% CI	
AI	65.87	62.96	69.83	161.00	155.66	166.03	0
PS	488.23	480	496	1051.65	1033.30	1071.21	0
MC (state 0)	29	29	29	4.81	4.04	6.12	P<= 0.001
MC (state 1)	9.67	9	12	3.31	2.69	4.15	P<= 0.001
MC (state 2)	16.85	16	22	6.12	5.06	8.02	P<= 0.001

*HPD CIs = highest posterior density confidence intervals (credible sets).

*state 0: vaccinated poultry; state 1: unvaccinated poultry; state 2: wild birds.

Table S5. Number of cumulative lineage transitions between wild birds and poultry populations with different vaccination statuses.

Dataset	Lineage transitions between hosts	Mean	95% HPD
Dataset R1	wild birds to vaccinated poultry populations	31	(22, 39)
	vaccinated poultry populations to wild birds	127	(118, 136)
	wild birds to unvaccinated poultry populations	263	(245, 280)
	unvaccinated poultry populations to wild birds	53	(38, 70)
Dataset R2	wild birds to vaccinated poultry populations	33	(24, 41)
	vaccinated poultry populations to wild birds	128	(118, 136)
	wild birds to unvaccinated poultry populations	243	(223, 259)
	unvaccinated poultry populations to wild birds	51	(34, 66)
Dataset R3	wild birds to vaccinated poultry populations	21	(13, 29)
	vaccinated poultry populations to wild birds	122	(113, 131)
	wild birds to unvaccinated poultry populations	211	(181, 241)
	unvaccinated poultry populations to wild birds	43	(23, 60)

Table S6. Segmented linear regression of nonsynonymous divergence in the Chinese poultry lineage from 1997 to 2019.

(nonsynonymous divergence ~ year)	Parameter	Estimate	Standard Error	95%CI
	Intercept	-3.3933	0.5621	
1996-2004	$slope_1$	0.0017	0.0002	(0.0011, 0.0023)
2005-2010	$slope_2$	0.0046	0.0002	(0.0041, 0.0052)
2010-2022	$slope_3$	0.0013	0.0002	(0.0010, 0.0017)

*adjusted R-squared: 0.9948; multiple R-Squared: 0.9961; residual standard error: 0.001294.

Table S7. Selection pressure in the HA gene of different host populations.

Host-specific lineages	cN (mean)	uN (mean)	cS (mean)	uS (mean)	dnds (mean)
Chinese poultry lineage	1651.50	2708.31	2899.66	1148.26	0.24
Bangladesh poultry lineage	162.32	380.10	425.67	165.38	0.17
Indonesia poultry lineage I	189.03	415.69	459.20	179.53	0.18
Indonesia poultry lineage II	254.15	551.89	589.73	229.27	0.18
late-wild bird lineage	498.40	1099.57	1203.66	468.89	0.18
early-wild bird lineage	229.63	545.6	614.62	230.28	0.16

*cN, the nonsynonymous substitution counts; uN, the unconditional nonsynonymous substitutions; cS, the synonymous substitution counts; uS, the unconditional synonymous substitutions.

Table S8. Positively-selected sites in the HA gene in resampled-Chinese poultry lineage.

**Lineage**	**Methods**			
	**RC**	**FEL (*p* < 0.1)**	**SLAC (*p* < 0.1)**	**FUBAR** (PP **> 0.9)**
Resampled-Chinese poultry lineage	61, 87, 136, 142, 154, 156, 157, 172, 178, 190, 205, 285, 289	61, 87*, 131, 142, 154*, 156, 157*, 172*, 190, 205, 216, 225	87, 154*, 157, 172, 205	87*, 131, 154*, 156*, 157*, 172, 205*

*Asterisks mark sites inferred to be under positively selected with posterior probability (PP) >0.95 and <0.05. FEL, Fixed Effects Likelihood. SLAC, Single Likelihood Ancestor Counting. FUBAR, Fast Unconstrained Bayesian AppRoximation. RC, renaissance counting method implemented in BEAST.

Table S9. Positively-selected sites in the HA gene among different host lineages inferred from *Dataset S1*.

**Lineage**	**FEL (*p* < 0.1)**	**SLAC (*p* < 0.1)**	**FUBAR (PP > 0.9)**
Chinese poultry lineage	3, 8*, 87*, 111*, 142, 145, 154*, 157, 171*, 172*, 185, 285, 291*, 325	3, 142*, 143, 154*, 157*, 172*, 185*, 285*	142*, 154*, 157*, 171, 285*
late-wild bird lineage	507	-	-
early-wild bird lineage	102*, 170*, 171	102, 107	102*, 170*, 171*

*Asterisks mark sites inferred to be under positively selected with posterior probability (PP) >0.95 and <0.05. FEL, Fixed Effects Likelihood. SLAC, Single Likelihood Ancestor Counting. FUBAR, Fast Unconstrained Bayesian AppRoximation.

Table S10. Positively-selected sites in the HA gene among different host lineages inferred from *Dataset S2*.

**Lineage**	**FEL (*p* < 0.1)**	**SLAC (*p* < 0.1)**	**FUBAR (PP > 0.9)**
Chinese poultry lineage	3, 8*, 87*, 111*, 142, 145, 154*, 157, 171*, 172*, 185, 285, 291*, 325	3, 8, 87, 111, 142, 145*, 154*, 157, 171*, 172*, 185*, 205, 338*	87, 154*, 157, 171*, 172
late-wild bird lineage	7, 185, 204, 509	-	-
early-wild bird lineage	102*, 113, 170*, 171	102*, 170	102*, 170*, 113, 171

*Asterisks mark sites inferred to be under positively selected with posterior probability (PP) >0.95 and <0.05. FEL, Fixed Effects Likelihood. SLAC, Single Likelihood Ancestor Counting. FUBAR, Fast Unconstrained Bayesian AppRoximation.

Table S11. The association between phylogeny and sampling population.

Statistic	observed			null			significance
	mean	lower 95% HPD CIs	upper 95% HPD CIs	mean	lower 95% HPD CIs	upper 95% HPD CIs	
AI	69.38	66.46	73.49	187.94	183.38	192.58	*P*=0
PS	529.69	521	538	1213.40	1199.97	1226.98	*P*=0
MC (state 0)	29	29	29	1.79	1.16	2.13	*P*<=0.001
MC (state 1)	14.22	14	15	3.24	2.55	4.07	*P*<=0.001
MC (state 2)	9.65	9	12	2.71	2.08	3.46	*P*<=0.001
MC (state 3)	7.90	6	10	1.88	1.27	2.17	*P*<=0.001
MC (state 4)	4.18	4	5	1.49	1	2	*P*<=0.001
MC (state 5)	6.75	6	9	1.47	1	2	*P*<=0.001
MC (state 6)	13.64	9	19	2.03	1.59	2.54	*P*<=0.001
MC (state 7)	16.85	16	22	6.12	5.07	8	*P*<=0.001

*HPD CIs = highest posterior density confidence intervals (credible sets).

*state 0: Bangladeshi poultry, state 1: Chinese poultry, state 2: European poultry, state 3: Indonesian poultry, state 4: Japanese poultry, state 5: Korean poultry, state 6: Vietnamese poultry, state 7: wild birds.

REFERENCES AND NOTES

1. T. Kuiken, R. Cromie, Protect wildlife from livestock diseases. *Science* **378**, 5 (2022).

2. E. F. Safety, I. Aznar, F. Baldinelli, A. Stoicescu, L. Kohnle, Annual report on surveillance for avian influenza in poultry and wild birds in Member States of the European Union in 2021. *EFSA J.* **20**, e07554 (2022).

3. C. Lebarbenchon, C. J. Feare, F. Renaud, F. Thomas, M. Gauthier-Clerc, Persistence of highly pathogenic avian influenza viruses in natural ecosystems. *Emerging Infect. Dis.* **16**, 1057–1062 (2010).

4. D. J. Alexander, An overview of the epidemiology of avian influenza. *Vaccine* **25**, 5637–5644 (2007).

5. B. Olsen, V. J. Munster, A. Wallensten, J. Waldenstrom, A. Osterhaus, R. A. M. Fouchier, Global patterns of influenza A virus in wild birds. *Science* **312**, 384–388 (2006).

6. H. Y. Tian, S. Zhou, L. Dong, T. P. Van Boeckel, Y. J. Cui, Y. R. Wu, B. Cazelles, S. Q. Huang, R. F. Yang, B. T. Grenfell, B. Xu, Avian influenza H5N1 viral and bird migration networks in Asia. *Proc. Natl. Acad. Sci. U.S.A.* **112**, 172–177 (2015).

7. G. G. Zhang, B. Y. Li, J. Raghwani, B. Vrancken, R. Jia, S. C. Hill, G. Fournie, Y. C. Cheng, Q. Q. Yang, Y. X. Wang, Z. M. Wang, L. Dong, O. G. Pybus, H. Y. Tian, Bidirectional movement of emerging H5N8 avian influenza viruses between Europe and Asia via migratory birds since early 2020. *Mol. Biol. Evol.* **40**, msad019 (2023).

8. H. Y. Tian, Y. J. Cui, L. Dong, S. Zhou, X. W. Li, S. Q. Huang, R. F. Yang, B. Xu, Spatial, temporal and genetic dynamics of highly pathogenic avian influenza A (H5N1) virus in China. *BMC Infect. Dis.* **15**, 54 (2015).

9. J. K. Taubenberger, J. C. Kash, Influenza virus evolution, host adaptation, and pandemic formation. *Cell Host Microbe* **7**, 440–451 (2010).

10. V. G. Dugan, R. Chen, D. J. Spiro, N. Sengamalay, J. Zaborsky, E. Ghedin, J. Nolting, D. E. Swayne, J. A. Runstadler, G. M. Happ, D. A. Senne, R. X. Wang, R. D. Slemons, E. C. Holmes, J. K. Taubenberger, The evolutionary genetics and emergence of avian influenza viruses in wild birds. *PLOS Pathog.* **4**, e1000076 (2008).

11. J. S. M. Peiris, M. D. de Jong, Y. Guan, Avian influenza virus (H5N1): A threat to human health. *Clin. Microbiol. Rev.* **20**, 243–267 (2007).

12. J. Pasick, Y. Berhane, T. Joseph, V. Bowes, T. Hisanaga, K. Handel, S. Alexandersen, Reassortant highly pathogenic influenza A H5N2 virus containing gene segments related to Eurasian H5N8 in British Columbia, Canada, 2014. *Sci. Rep.* **5**, 9484 (2015).

13. K. Bertran, D. E. Swayne, M. J. Pantin-Jackwood, D. R. Kapczynski, E. Spackman, D. L. Suarez, Lack of chicken adaptation of newly emergent Eurasian H5N8 and reassortant H5N2 high pathogenicity avian influenza viruses in the US is consistent with restricted poultry outbreaks in the Pacific flyway during 2014-2015. *Virology* **494**, 190–197 (2016).

14. X. Y. Li, P. F. Cui, X. Y. Zeng, Y. P. Jiang, Y. B. Li, J. X. Yang, Y. D. Pan, X. X. Gao, C. H. Zhao, J. H. Wang, K. Wang, G. H. Deng, J. Guo, Characterization of avian influenza H5N3 reassortants isolated from migratory waterfowl and domestic ducks in China from 2015 to 2018. *Transbound. Emerging. Dis.* **66**, 2605–2610 (2019).

15. S. J. Yeo, V. T. Hoang, T. B. Duong, N. M. Nguyen, H. T. Tuong, M. Azam, H. W. Sung, H. Park, Emergence of a novel reassortant H5N3 avian influenza virus in Korean mallard ducks in 2018. *Intervirology* **65**, 1–16 (2022).

16. M. Gu, W. Liu, Y. Cao, D. Peng, X. Wang, H. Wan, G. Zhao, Q. Xu, W. Zhang, Q. Song, Y. Li, X. Liu, Novel reassortant highly pathogenic avian influenza (H5N5) viruses in domestic ducks, China. *Emerg. Infect. Dis.* **17**, 1060–1063 (2011).

17. Y. M. Kang, H. K. Cho, H. M. Kim, C. H. Lee, D. Y. Kim, S. H. Choi, M. H. Lee, H. M. Kang, Protection of layers and breeders against homologous or heterologous HPAIv by vaccines from Korean national antigen bank. *Sci. Rep.* **10**, 9436 (2020).

18. J. P. Villanueva-Cabezas, M. J. C. Coppo, P. A. Durr, J. McVernon, Vaccine efficacy against Indonesian Highly Pathogenic Avian Influenza H5N1: Systematic review and meta-analysis. *Vaccine* **35**, 4859–4869 (2017).

19. E. M. Hill, T. House, M. S. Dhingra, W. Kalpravidh, S. Morzaria, M. G. Osmani, E. Brum, M. Yamage, M. A. Kalam, D. J. Prosser, J. Y. Takekawa, X. M. Xiao, M. Gilbert, M. J. Tildesley, The impact of surveillance and control on highly pathogenic avian influenza outbreaks in poultry in Dhaka division, Bangladesh. *PLOS Comput. Biol.* **14**, e1006439 (2018).

20. H. T. T. Hoang, C. H. Nguyen, N. T. T. Nguyen, A. D. Pham, H. T. T. Nguyen, T. H. Le, H. X. Tran, H. H. Chu, N. T. Nguyen, Immunization with the H5N1 recombinant vaccine candidate induces high protection in chickens against Vietnamese highly pathogenic avian influenza virus strains. *Vaccines* **8**, 159 (2020).

21. X. Zeng, G. Tian, J. Shi, G. Deng, C. Li, H. Chen, Vaccination of poultry successfully eliminated human infection with H7N9 virus in China. *Sci. China Life Sci.* **61**, 1465–1473 (2018).

22. D. E. Swayne, Impact of vaccines and vaccination on global control of avian influenza. *Avian Dis.* **56**, 818–828 (2012).

23. R. Parvin, M. Nooruzzaman, C. K. Kabiraj, J. A. Begum, E. H. Chowdhury, M. R. Islam, T. Harder, Controlling avian influenza virus in Bangladesh: Challenges and recommendations. *Viruses* **12**, 751 (2020).

24. S. Liu, Q. Y. Zhuang, S. C. Wang, W. M. Jiang, J. H. Jin, C. Peng, G. Y. Hou, J. P. Li, J. M. Yu, X. H. Yu, H. L. Liu, S. F. Sun, L. P. Yuan, J. M. Chen, Control of avian influenza in China: Strategies and lessons. *Transbound. Emerg. Dis.* **67**, 1463–1471 (2020).

25. Z. Sun, J. Wang, Z. Huang, Assessment of China's H5N1 routine vaccination strategy. *Sci. Rep.* **7**, 46441 (2017).

26. J. Wu, C. Ke, E. H. Y. Lau, Y. Song, K. L. Cheng, L. Zou, M. Kang, T. Song, M. Peiris, H.-L. Yen, Influenza H5/H7 virus vaccination in poultry and reduction of zoonotic infections, Guangdong Province, China, 2017–18. *Emerg. Infect. Dis.* **25**, 116–118 (2019).

27. L. L. Liu, X. Y. Zeng, P. C. Chen, G. H. Deng, Y. B. Li, J. Z. Shi, C. Y. Gu, H. H. Kong, Y. Suzuki, Y. P. Jiang, G. B. Tian, H. L. Chen, Characterization of clade 7.2 H5 avian influenza viruses that continue to circulate in chickens in China. *J. Virol.* **90**, 9797–9805 (2016).

28. W. Song, J. Guo, X. Ni, J. Wu, W. Xia, F. He, X. Wang, G. Fan, K. Zhou, Y. Wu, S. Chen, H. Chen, Changes of avian influenza virus subtypes before and after vaccination in live poultry in Nanchang, China from 2016 to 2019. *Microbes Infect.* **23**, 104848 (2021).

29. S. Iwami, T. Suzuki, Y. Takeuchi, Paradox of vaccination: Is vaccination really effective against avian flu epidemics? *PLOS ONE* **4**, e4915 (2009).

30. Z. Wang, W. Jiang, S. Liu, G. Hou, J. Li, Z. Wang, J. Chen, Increased substitution rate in H5N1 avian influenza viruses during mass vaccination of poultry. *Chin. Sci. Bull.* **57**, 2419–2424 (2012).

31. C. W. Lee, D. A. Senne, D. L. Suarez, Effect of vaccine use in the evolution of Mexican lineage H5N2 avian influenza virus. *J. Virol.* **78**, 8372–8381 (2004).

32. G. Cattoli, A. Fusaro, I. Monne, F. Coven, T. Joannis, H. S. A. El-Hamid, A. A. Hussein, C. Cornelius, N. M. Amarin, M. Mancin, E. C. Holmes, I. Capua, Evidence for differing evolutionary dynamics of A/H5N1 viruses among countries applying or not applying avian influenza vaccination in poultry. *Vaccine* **29**, 9368–9375 (2011).

33. J. H. Kwon, D. H. Lee, M. F. Criado, L. Killmaster, M. Z. Ali, M. Giasuddin, M. A. Samad, M. R. Karim, M. Hasan, E. Brum, T. Nasrin, D. E. Swayne, Genetic evolution and transmission dynamics of clade 2.3.2.1a highly pathogenic avian influenza A/H5N1 viruses in Bangladesh. *Virus Evol.* **6**, veaa046 (2020).

34. M. Ghafari, L. du Plessis, J. Raghwani, S. Bhatt, B. Xu, O. G. Pybus, A. Katzourakis, Purifying selection determines the short-term time dependency of evolutionary rates in SARS-CoV-2 and pH1N1 influenza. *Mol. Biol. Evol.* **39**, msac009 (2022).

35. M. Fourment, E. C. Holmes, Avian influenza virus exhibits distinct evolutionary dynamics in wild birds and poultry. *BMC Evol. Biol.* **15**, 120 (2015).

36. S. Bhatt, E. C. Holmes, O. G. Pybus, The genomic rate of molecular adaptation of the human influenza A virus. *Mol. Biol. Evol.* **28**, 2443–2451 (2011).

37. P. Lemey, V. N. Minin, F. Bielejec, S. L. Kosakovsky Pond, M. A. Suchard, A counting renaissance: Combining stochastic mapping and empirical Bayes to quickly detect amino acid sites under positive selection. *Bioinformatics* **28**, 3248–3256 (2012).

38. X. Qiu, V. R. Duvvuri, J. B. Gubbay, R. J. Webby, G. Kayali, J. Bahl, Lineage-specific epitope profiles for HPAI H5 pre-pandemic vaccine selection and evaluation. *Influenza Other Respi. Viruses* **11**, 445–456 (2017).

39. M. Qian, H. Hu, T. Zuo, G. Wang, L. Zhang, P. Zhou, Unraveling of a neutralization mechanism by two human antibodies against conserved epitopes in the globular head of H5 hemagglutinin. *J. Virol.* **87**, 3571–3577 (2013).

40. H. R. Haghighi, L. R. Read, S. M. Haeryfar, S. Behboudi, S. Sharif, Identification of a dual-specific T cell epitope of the hemagglutinin antigen of an H5 avian influenza virus in chickens. *PLOS ONE* **4**, e7772 (2009).

41. J. King, T. Harder, F. J. Conraths, M. Beer, A. Pohlmann, The genetics of highly pathogenic avian influenza viruses of subtype H5 in Germany, 2006–2020. *Transbound. Emerg. Dis.* **68**, 1136–1150 (2021).

42. The Global Consortium for H5N8 and Related Influenza Viruses, Role for migratory wild birds in the global spread of avian influenza H5N8. *Science* **354**, 213–217 (2016).

43. Q. Q. Yang, X. Zhao, P. Lemey, M. A. Suchard, Y. H. Bi, W. F. Shi, D. Liu, W. B. Qi, G. G. Zhang, N. C. Stenseth, O. G. Pybus, H. Y. Tian, Assessing the role of live poultry trade in community-structured transmission of avian influenza in China. *Proc. Natl. Acad. Sci. U.S.A.* **117**, 5949–5954 (2020).

44. V. Caliendo, L. Leijten, M. W. G. van de Bildt, M. J. Poen, A. Kok, T. Bestebroer, M. Richard, R. A. M. Fouchier, T. Kuiken, Long-term protective effect of serial infections with H5N8 highly pathogenic avian influenza virus in wild ducks. *J. Virol.* **96**, e01233–01222 (2022).

45. S. Koethe, L. Ulrich, R. Ulrich, S. Amler, A. Graaf, T. C. Harder, C. Grund, T. C. Mettenleiter, F. J. Conraths, M. Beer, A. Globig, Modulation of lethal HPAIV H5N8 clade 2.3.4.4B infection in AIV pre-exposed mallards. *Emerg. Microbes Infect.* **9**, 180–193 (2020).

46. S. C. Hill, R. J. Manvell, B. Schulenburg, W. Shell, P. S. Wikramaratna, C. Perrins, B. C. Sheldon, I. H. Brown, O. G. Pybus, Antibody responses to avian influenza viruses in wild birds broaden with age. *Proc. R. Soc. London Ser. B* **283**, 20162159 (2016).

47. J. H. Verhagen, U. Hofle, G. van Amerongen, M. van de Bildt, F. Majoor, R. A. M. Fouchier, T. Kuiken, Long-term effect of serial infections with H13 and H16 low-pathogenic avian influenza viruses in black-headed gulls. *J. Virol.* **89**, 11507–11522 (2015).

48. J. P. Bird, R. Martin, H. R. Akcakaya, J. Gilroy, I. J. Burfield, S. T. Garnett, A. Symes, J. Taylor, C. H. Sekercioglu, S. H. M. Butchart, Generation lengths of the world's birds and their implications for extinction risk. *Conserv. Biol.* **34**, 1252–1261 (2020).

49. Y. Zhang, P. Cui, J. Shi, Y. Chen, X. Zeng, Y. Jiang, G. Tian, C. Li, H. Chen, H. Kong, G. Deng, Key amino acid residues that determine the antigenic properties of highly pathogenic H5 influenza viruses bearing the clade 2.3.4.4 hemagglutinin gene. *Viruses* **15**, 2249 (2023).

50. W. M. Jiang, C. X. Dong, S. Liu, C. Peng, X. Yin, S. B. Liang, L. Zhang, J. P. Li, X. H. Yu, Y. Li, J. J. Wang, G. Y. Hou, Z. Zeng, H. L. Liu, Emerging novel reassortant influenza A (H5N6) viruses in poultry and humans, china, 2021. *Emerg. Infect. Dis.* **28**, 1064–1066 (2022).

51. J. Zhang, H. Ye, Y. Liu, M. Liao, W. Qi, Resurgence of H5N6 avian influenza virus in 2021 poses new threat to public health. *Lancet Microbe* **3**, e558–e558 (2022).

52. K. Ciminski, G. Chase, M. Schwemmle, M. Beer, Advocating a watch-and-prepare approach with avian influenza. *Nat. Microbiol.* **8**, 1603–1605 (2023).

53. M. Kozlov, US will vaccinate birds against avian flu for first time. *Nature* **618**, 220–221 (2023).

54. M. Klaassen, M. Wille, The plight and role of wild birds in the current bird flu panzootic. *Nat. Ecol. Evol.* **7**, 1541–1542 (2023).

55. M. Wille, I. G. Barr, Resurgence of avian influenza virus. *Science* **376**, 459–460 (2022).

56. I. Sitaras, X. Rousou, D. Kalthoff, M. Beer, B. Peeters, M. C. M. de Jong, Role of vaccination-induced immunity and antigenic distance in the transmission dynamics of highly pathogenic avian influenza H5N1. *J. R. Soc. Interface* **13**, 20150976 (2016).

57. P. G. T. Walker, S. Cauchemez, R. Metras, D. H. Dung, D. Pfeiffer, A. C. Ghani, A Bayesian approach to quantifying the effects of mass poultry vaccination upon the spatial and temporal dynamics of H5N1 in Northern Vietnam. *PLOS Comput. Biol.* **6**, e1000683 (2010).

58. P. F. Cui, X. Y. Zeng, X. Y. Li, Y. B. Li, J. Z. Shi, C. H. Zhao, Z. Y. Qu, Y. W. Wang, J. Guo, W. L. Gu, Q. Ma, Y. C. Zhang, W. P. Lin, M. H. Li, J. M. Tian, D. X. Wang, X. Xing, Y. J. Liu, S. X. Pan, Y. P. Zhang, H. M. Bao, L. L. Liu, G. B. Tian, C. J. Li, G. H. Deng, H. L. Chen, Genetic and biological characteristics of the globally circulating H5N8 avian influenza viruses and the protective efficacy offered by the poultry vaccine currently used in China. *Sci. China Life Sci.* **65**, 795–808 (2022).

59. O. N. Poetri, A. Bouma, S. Murtini, I. Claassen, G. Koch, R. D. Soejoedono, J. A. Stegeman, M. van Boven, An inactivated H5N2 vaccine reduces transmission of highly pathogenic H5N1 avian influenza virus among native chickens. *Vaccine* **27**, 2864–2869 (2009).

60. C. Terregino, A. Toffan, F. Cilloni, I. Monne, E. Bertoli, L. Castellanos, N. Amarin, M. Mancin, I. Capua, Evaluation of the protection induced by avian influenza vaccines containing a 1994

Mexican H5N2 LPAI seed strain against a 2008 Egyptian H5N1 HPAI virus belonging to clade 2.2.1 by means of serological and in vivo tests. *Avian Pathol.* **39**, 215–222 (2010).

61. T. M. Ellis, C. Leung, M. K. W. Chow, L. A. Bissett, W. Wong, Y. Guan, J. S. M. Peiris, Vaccination of chickens against H5N1 avian influenza in the face of an outbreak interrupts virus transmission. *Avian Pathol.* **33**, 405–412 (2004).

62. R. J. S. Magalhaes, D. U. Pfeiffer, J. Otte, Evaluating the control of HPAIV H5N1 in Vietnam: Virus transmission within infected flocks reported before and after vaccination. *BMC Vet. Res.* **6**, 31 (2010).

63. A. Islam, S. Munro, M. M. Hassan, J. H. Epstein, M. Klaassen, The role of vaccination and environmental factors on outbreaks of high pathogenicity avian influenza H5N1 in Bangladesh. *One Health* **17**, 100655 (2023).

64. O. Poetri, A. Bouma, I. Claassen, G. Koch, R. Soejoedono, A. Stegeman, M. van Boven, A single vaccination of commercial broilers does not reduce transmission of H5N1 highly pathogenic avian influenza. *Vet. Res.* **42**, 74 (2011).

65. A. F. Read, S. J. Baigent, C. Powers, L. B. Kgosana, L. Blackwell, L. P. Smith, D. A. Kennedy, S. W. Walkden-Brown, V. K. Nair, Imperfect vaccination can enhance the transmission of highly virulent pathogens. *PLOS Biol.* **13**, e1002198 (2015).

66. S. Gandon, M. J. Mackinnon, S. Nee, A. F. Read, Imperfect vaccines and the evolution of pathogen virulence. *Nature* **414**, 751–756 (2001).

67. B. T. Grenfell, O. G. Pybus, J. R. Gog, J. L. N. Wood, J. M. Daly, J. A. Mumford, E. C. Holmes, Unifying the epidemiological and evolutionary dynamics of pathogens. *Science* **303**, 327–332 (2004).

68. J. Raghwani, S. Bhatt, O. G. Pybus, Faster adaptation in smaller populations: Counterintuitive evolution of HIV during childhood infection. *PLOS Comput. Biol.* **12**, e1004694 (2016).

69. J. Guo, W. T. Song, X. S. Ni, W. Liu, J. W. Wu, W. Xia, X. F. Zhou, W. Wang, F. L. He, X. Wang, G. Y. Fan, K. Zhou, H. Y. Chen, S. G. Chen, Pathogen change of avian influenza virus in the live poultry market before and after vaccination of poultry in southern China. *Virol. J.* **18**, 213 (2021).

70. W. Cheng, K. C. Chong, T. Lau, X. X. Wang, Z. Yu, S. L. Liu, M. Wang, J. R. Pan, E. F. Chen, Comparison of avian influenza virus contamination in the environment before and after massive poultry H5/H7 vaccination in Zhejiang Province, China. *Open Forum Infect. Dis.* **6**, ofz197 (2019).

71. K. Katoh, D. M. Standley, MAFFT multiple sequence alignment software Version 7: Improvements in performance and usability. *Mol. Biol. Evol.* **30**, 772–780 (2013).

72. B. J. Baker, V. De Anda, K. W. Seitz, N. Dombrowski, A. E. Santoro, K. G. Lloyd, Diversity, ecology and evolution of Archaea. *Nat. Microbiol.* **5**, 887–900 (2020).

73. D. P. Martin, B. Murrell, M. Golden, A. Khoosal, B. Muhire, RDP4: Detection and analysis of recombination patterns in virus genomes. *Virus Evol.* **1**, vev003 (2015).

74. I. E. Kiwelu, V. Novitsky, L. Margolin, J. Baca, R. Manongi, N. Sam, J. Shao, M. F. McLane, S. H. Kapiga, M. Essex, Frequent intra-subtype recombination among HIV-1 circulating in Tanzania. *PLOS ONE* **8**, e71131 (2013).

75. E. Y. Koh, J. Ong, Y. F. Wang, X. Toh, C. J. Fernandez, T. Q. Huangfu, R. N. Hall, S. Toh, K. Lim, W. Sng, H. P. Lim, K. L. Ho, S. F. Chang, H. H. Yap, Rabbit haemorrhagic disease virus 2 from Singapore 2020 outbreak revealed an Australian recombinant variant. *Virus Evol.* **9**, vead029 (2023).

76. M. N. Price, P. S. Dehal, A. P. Arkin, FastTree 2—Approximately maximum-likelihood trees for large alignments. *PLOS ONE* **5**, e9490 (2010).

77. A. Rambaut, T. T. Lam, L. M. Carvalho, O. G. Pybus, Exploring the temporal structure of heterochronous sequences using TempEst (formerly Path-O-Gen). *Virus Evol.* **2**, vew007 (2016).

78. A. Kalkauskas, U. Perron, Y. X. Sun, N. Goldman, G. Baele, S. Guindon, N. De Maio, Sampling bias and model choice in continuous phylogeography: Getting lost on a random walk. *PLOS Comput. Biol.* **17**, e1008561 (2021).

79. P. Lemey, S. L. Hong, V. Hill, G. Baele, C. Poletto, V. Colizza, A. O'Toole, J. T. McCrone, K. G. Andersen, M. Worobey, M. I. Nelson, A. Rambaut, M. A. Suchard, Accommodating individual travel history and unsampled diversity in Bayesian phylogeographic inference of SARS-CoV-2. *Nat. Commun.* **11**, 5110 (2020).

80. M. Escalera-Zamudio, M. Golden, B. Gutiérrez, J. Thézé, J. R. Keown, L. Carrique, T. A. Bowden, O. G. Pybus, Parallel evolution in the emergence of highly pathogenic avian influenza A viruses. *Nat. Commun.* **11**, 5511 (2020).

81. W.-T. He, L. Wang, Y. Zhao, N. Wang, G. Li, M. Veit, Y. Bi, G. F. Gao, S. Su, Adaption and parallel evolution of human-isolated H5 avian influenza viruses. *J. Infect.* **80**, 630–638 (2020).

82. M. A. Suchard, P. Lemey, G. Baele, D. L. Ayres, A. J. Drummond, A. Rambaut, Bayesian phylogenetic and phylodynamic data integration using BEAST 1.10. *Virus Evol.* **4**, vey016 (2018).

83. D. L. Ayres, A. Darling, D. J. Zwickl, P. Beerli, M. T. Holder, P. O. Lewis, J. P. Huelsenbeck, F. Ronquist, D. L. Swofford, M. P. Cummings, A. Rambaut, M. A. Suchard, BEAGLE: An application programming interface and high-performance computing library for statistical phylogenetics. *Syst. Biol.* **61**, 170–173 (2012).

84. V. Hill, G. Baele, Bayesian estimation of past population dynamics in BEAST 1.10 using the Skygrid coalescent model. *Mol. Biol. Evol.* **36**, 2620–2628 (2019).

85. P. Lemey, A. Rambaut, T. Bedford, N. Faria, F. Bielejec, G. Baele, C. A. Russell, D. J. Smith, O. G. Pybus, D. Brockmann, M. A. Suchard, Unifying viral genetics and human transportation data to predict the global transmission dynamics of human influenza H3N2. *PLOS Pathog.* **10**, e1003932 (2014).

86. C. J. Edwards, M. A. Suchard, P. Lemey, J. J. Welch, I. Barnes, T. L. Fulton, R. Barnett, T. C. O'Connell, P. Coxon, N. Monaghan, C. E. Valdiosera, E. D. Lorenzen, E. Willerslev, G. F. Baryshnikov, A. Rambaut, M. G. Thomas, D. G. Bradley, B. Shapiro, Ancient hybridization and an Irish origin for the modern polar bear matriline. *Curr. Biol.* **21**, 1251–1258 (2011).

87. G. Dudas, L. M. Carvalho, A. Rambaut, T. Bedford, MERS-CoV spillover at the camel-human interface. *eLife* **7**, e31257 (2018).

88. G. C. Yu, D. K. Smith, H. C. Zhu, Y. Guan, T. T. Y. Lam, GGTREE: An R package for visualization and annotation of phylogenetic trees with their covariates and other associated data. *Methods Ecol. Evol.* **8**, 28–36 (2017).

89. P. T. Nguyen, R. Kant, F. van den Broeck, M. T. Suvanto, H. Alburkat, J. Virtanen, E. Ahvenainen, R. Castren, S. L. Hong, G. Baele, M. J. Ahava, H. Jarva, S. T. Jokiranta, H. Kallio-Kokko, E. Kekaelaeinen, V. Kirjavainen, E. Kortela, S. Kurkela, M. Lappalainen, H. Liimatainen, M. A. Suchard, S. Hannula, P. Ellonen, T. Sironen, P. Lemey, O. Vapalahti, T. Smura, The phylodynamics of SARS-CoV-2 during 2020 in Finland. *Commun. Med.* **2**, 65 (2022).

90. J. Parker, A. Rambaut, O. G. Pybus, Correlating viral phenotypes with phylogeny: Accounting for phylogenetic uncertainty. *Infect. Genet. Evol.* **8**, 239–246 (2008).

91. B. Murrell, S. Moola, A. Mabona, T. Weighill, D. Sheward, S. L. Kosakovsky Pond, K. Scheffler, FUBAR: A fast, unconstrained bayesian approximation for inferring selection. *Mol. Biol. Evol.* **30**, 1196–1205 (2013).

92. S. L. Kosakovsky Pond, S. D. Frost, Not so different after all: A comparison of methods for detecting amino acid sites under selection. *Mol. Biol. Evol.* **22**, 1208–1222 (2005).

93. S. L. K. Pond, A. F. Y. Poon, R. Velazquez, S. Weaver, N. L. Hepler, B. Murrell, S. D. Shank, B. R. Magalis, D. Bouvier, A. Nekrutenko, S. Wisotsky, S. J. Spielman, S. D. W. Frost, S. V. Muse, HyPhy 2.5—A customizable platform for evolutionary hypothesis testing using phylogenies. *Mol. Biol. Evol.* **37**, 295–299 (2020).

94. K. E. Kistler, T. Bedford, Evidence for adaptive evolution in the receptor-binding domain of seasonal coronaviruses OC43 and 229e. *eLife* **10**, e64509 (2021).

95. H. Ye, E. R. Deyle, L. J. Gilarranz, G. Sugihara, Distinguishing time-delayed causal interactions using convergent cross mapping. *Sci. Rep.* **5**, 14750 (2015).

96. M. Gilbert, G. Nicolas, G. Cinardi, T. P. Van Boeckel, S. O. Vanwambeke, G. Wint, T. P. Robinson, Global distribution data for cattle, buffaloes, horses, sheep, goats, pigs, chickens and ducks in 2010. *PLOS Comput. Biol.* **5**, 1–11 (2018).

97. J. Shi, X. Zeng, P. Cui, C. Yan, H. Chen, Alarming situation of emerging H5 and H7 avian influenza and effective control strategies. *Emerg. Microbes Infect.* **12**, 2155072 (2023).

98. X. Zeng, G. Tian, H. Chen, Progress in development and application of H5/H7 avian influenza vaccines in China. *Sci. Sin.* **53**, 1700–1712 (2023).

99. C. C. Tran, J. F. Yanagida, S. Saksena, J. Fox, An alternative vaccination approach for the prevention of highly pathogenic avian influenza subtype H5N1 in the Red River Delta, Vietnam—A geospatial-based cost-effectiveness analysis. *Vet. Sci.* **3**, 6 (2016).

100. D. H. Chu, in Vietnam Country Report on Vaccination for Avian Influenza (AI) (World Organisation for Animal Health, 2017).

101. S. Tarigan, M. H. Wibowo, R. Indriani, S. Sumarningsih, S. Artanto, S. Idris, P. A. Durr, W. Asmara, E. Ebrahimie, M. A. Stevenson, J. Ignjatovic, Field effectiveness of highly pathogenic avian influenza H5N1 vaccination in commercial layers in Indonesia. *PLOS ONE* **13**, e0190947 (2018).

102. D. E. Swayne, D. L. Suarez, E. Spackman, S. Jadhao, G. Dauphin, M. Kim-Torchetti, J. McGrane, J. Weaver, P. Daniels, F. Wong, P. Selleck, A. Wiyono, R. Indriani, Y. Yupiana, E. S. Siregar, T. Prajitno, D. Smith, R. Fouchier, Antibody titer has positive predictive value for vaccine protection against challenge with natural antigenic-drift variants of H5N1 high-pathogenicity avian influenza viruses from indonesia. *J. Virol.* **89**, 3746–3762 (2015).

103. Q. Yang, B. Wang, P. Lemey, L. Dong, T. Mu, R. A. Wiebe, F. Guo, N. S. Trovao, S. W. Park, N. Lewis, J. L. H. Tsui, S. Bajaj, Y. Cheng, L. Yang, Y. Haba, B. Li, G. Zhang, O. G. Pybus, H. Tian, B. Grenfell, Synchrony of bird migration with global dispersal of avian influenza reveals exposed bird orders. *Nat. Commun.* **15**, 1126 (2024).
